# Supplementary material for: Pre-operative stereotactic radiosurgery and peri-operative dexamethasone for resectable brain metastases: a two-arm pilot study evaluating clinical outcomes and immunological correlates
Source: Nat Commun. 2024 Oct 14;15:8854. doi: 10.1038/s41467-024-53034-6 (PMC11473782; doi:10.1038/s41467-024-53034-6)
Supplement: Supplementary file 1 — Supplementary Information [file 41467_2024_53034_MOESM1_ESM.pdf]

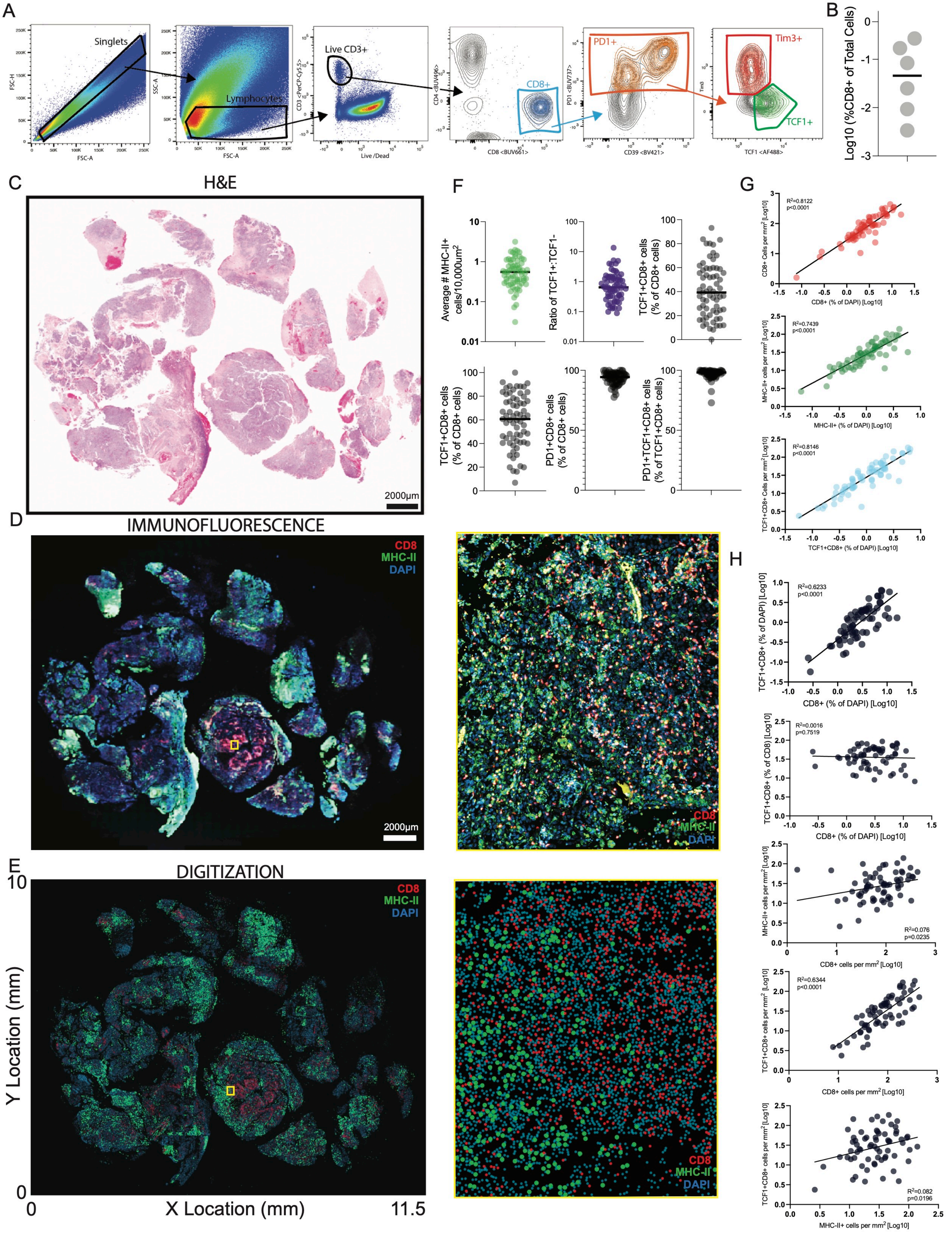

A
Singlets
Lymphocytes
Live CD3+
CD8+
PD1+
Tim3+
TCF1+
FSC-A
SSC-A
Live /Dead
CD8 <BUV661>
CD39 <BV421>
TCF1 <AF488>
B
Log10 (%CD8+ of Total Cells)
C
H&E
2000µm
D
IMMUNOFLUORESCENCE
CD8
MHC-II
DAPI
2000µm
E
DIGITIZATION
Y Location (mm)
X Location (mm)
11.5
F
Average # MHC-II+ cells/10,000um2
Ratio of TCF1+:TCF1-
TCF1+CD8+ cells (% of CD8+ cells)
PD1+CD8+ cells (% of CD8+ cells)
PD1+TCF1+CD8+ cells (% of TCF1+CD8+ cells)
G
R2=0.8122 p<0.0001
CD8+ (% of DAPI) [Log10]
R2=0.7439 p<0.0001
MHC-II+ (% of DAPI) [Log10]
R2=0.8146 p<0.0001
TCF1+CD8+ (% of DAPI) [Log10]
H
R2=0.6233 p<0.0001
TCF1+CD8+ (% of DAPI) [Log10]
CD8+ (% of DAPI) [Log10]
R2=0.0016 p=0.7519
TCF1+CD8+ (% of CD8) [Log10]
R2=0.076 p=0.0235
CD8+ cells per mm2 [Log10]
R2=0.6344 p<0.0001
R2=0.082 p=0.0196
MHC-II+ cells per mm2 [Log10]

Supplementary Figure 1. A) Flow cytometry gating strategy. **B)** Frequency of CD8 T cells by flow cytometry (n=6). **C)** Whole slide H&E image of BrM. **D)** Representative three-color immunofluorescence staining with region of interest shown in yellow box, with corresponding region of interest on the right. **E)** Digitization of the slide with region of interest shown, with corresponding region of interest on the right. **F)** Quantification of number of TCF1+ cells per mm^2, average number of MHC-II+ cells per $10{,}000um^2$, ratio of TCF1+ to TCF1- cells, percentage of TCF1+ or TCF1- of CD8 cells, percentage of CD8 cells that are PD1+, and percentage of TCF1+ CD8 cells that are PD1+. **G)** Validation of quantification methods, demonstrating correlation between a given cell count per mm^2 and respective proportion of total cells. **H**) Correlation between percent CD8 of total cells and percent TCF1+ CD8 of total cells or TCF1+ CD8 of CD8 cells, correlation between CD8 cells per mm^2 and MHC-II+ cells per mm^2, correlation between CD8 cells per mm^2 and TCF1+ CD8 cells per mm^2, correlation between MHC-II+ cells per mm^2 and TCF1+ CD8 cells per mm^2. For F-H, n=67 for each plot. Source data are provided as a Source Data file.

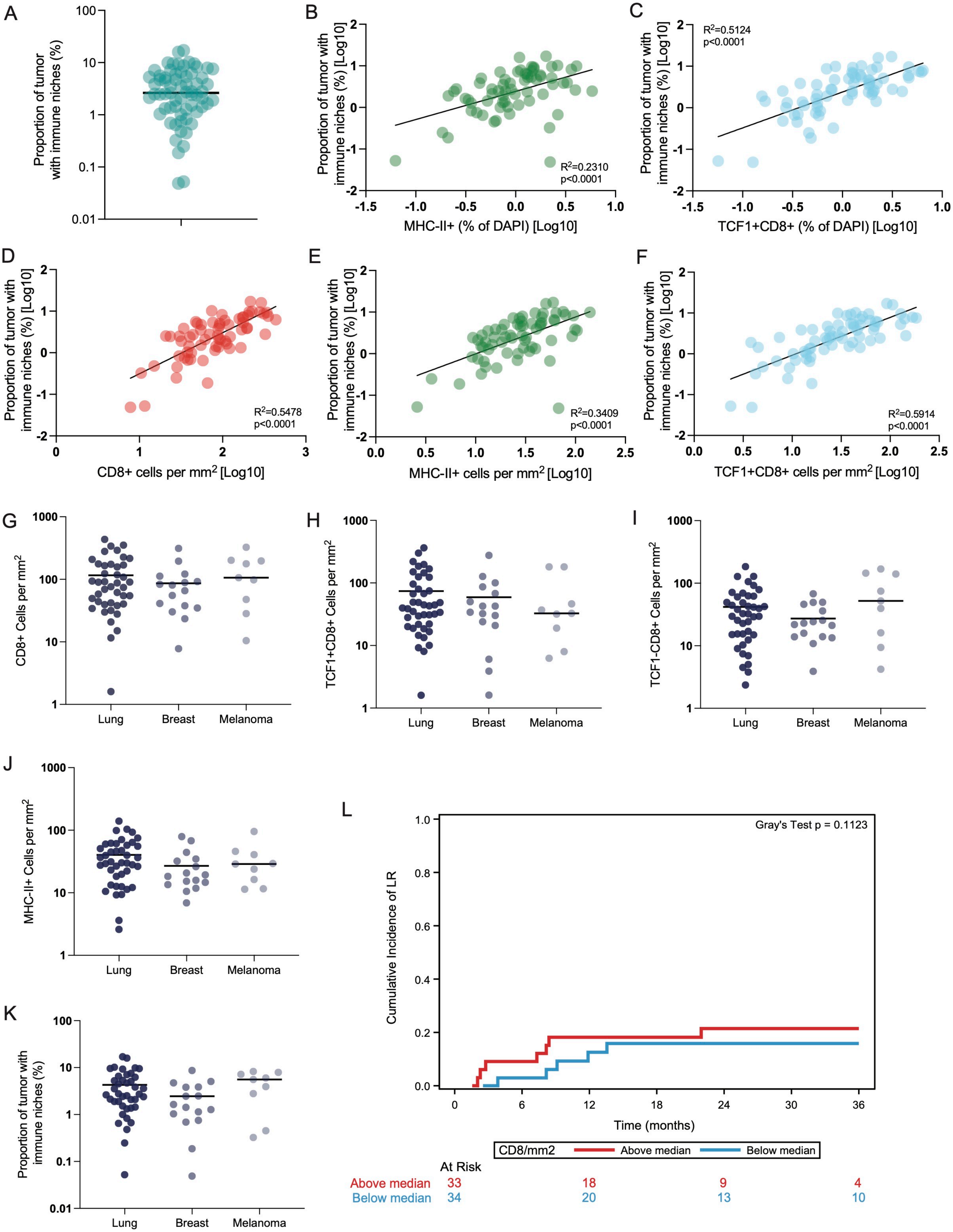
A
Proportion of tumor with immune niches (%)
100
10
1
0.1
0.01
B
Proportion of tumor with immune niches (%) [Log10]
MHC-II+ (% of DAPI) [Log10]
R2=0.2310
p<0.0001
C
TCF1+CD8+ (% of DAPI) [Log10]
R2=0.5124
p<0.0001
D
CD8+ cells per mm2 [Log10]
R2=0.5478
p<0.0001
E
MHC-II+ cells per mm2 [Log10]
R2=0.3409
p<0.0001
F
TCF1+CD8+ cells per mm2 [Log10]
R2=0.5914
p<0.0001
G
CD8+ Cells per mm2
H
TCF1+CD8+ Cells per mm2
I
TCF1-CD8+ Cells per mm2
J
MHC-II+ Cells per mm2
K
Proportion of tumor with immune niches (%)
Lung
Breast
Melanoma
L
Gray's Test p = 0.1123
Cumulative Incidence of LR
Time (months)
0
6
12
18
24
30
36
CD8/mm2
Above median
Below median
At Risk
Above median 33 18 9 4
Below median 34 20 13 10

Supplementary Figure 2. A) Quantification of the proportion of tumors occupied by immune niches (local 100um x 100um cellular neighborhoods where TCF1+ CD8 T cells and MHC-II+ cells co-localize). **B)** Correlation between MHC-II+ cell frequency and proportion of tumors with immune niches. **C)** Correlation between TCF1+ CD8 cell frequency and proportion of tumors with immune niches. **D)** Correlation between CD8 cells per mm^2 and proportion of tumors with immune niches. **E)** Correlation between MHC-II+ cells per mm^2 and proportion of tumors with immune niches. **F)** Correlation between TCF1+ CD8 cells per mm^2 and proportion of tumors with immune niches. **G-K)** Comparison of immune infiltrate across tumor histology by cell type: **G)** CD8 per mm^2, **H)** TCF1+ cells per mm^2, **I)** TCF1- cells per mm^2, **J)** MHC-II+ cells per mm^2 and **K)** proportion of immune niches. A-F, n=67 for each plot. G-K, n=42 lung, n=16 breast, n=9 melanoma. **L)** Cumulative incidence curve showing no difference in local control between high vs. low CD8 T cell per mm^2. Source data are provided as a Source Data file.

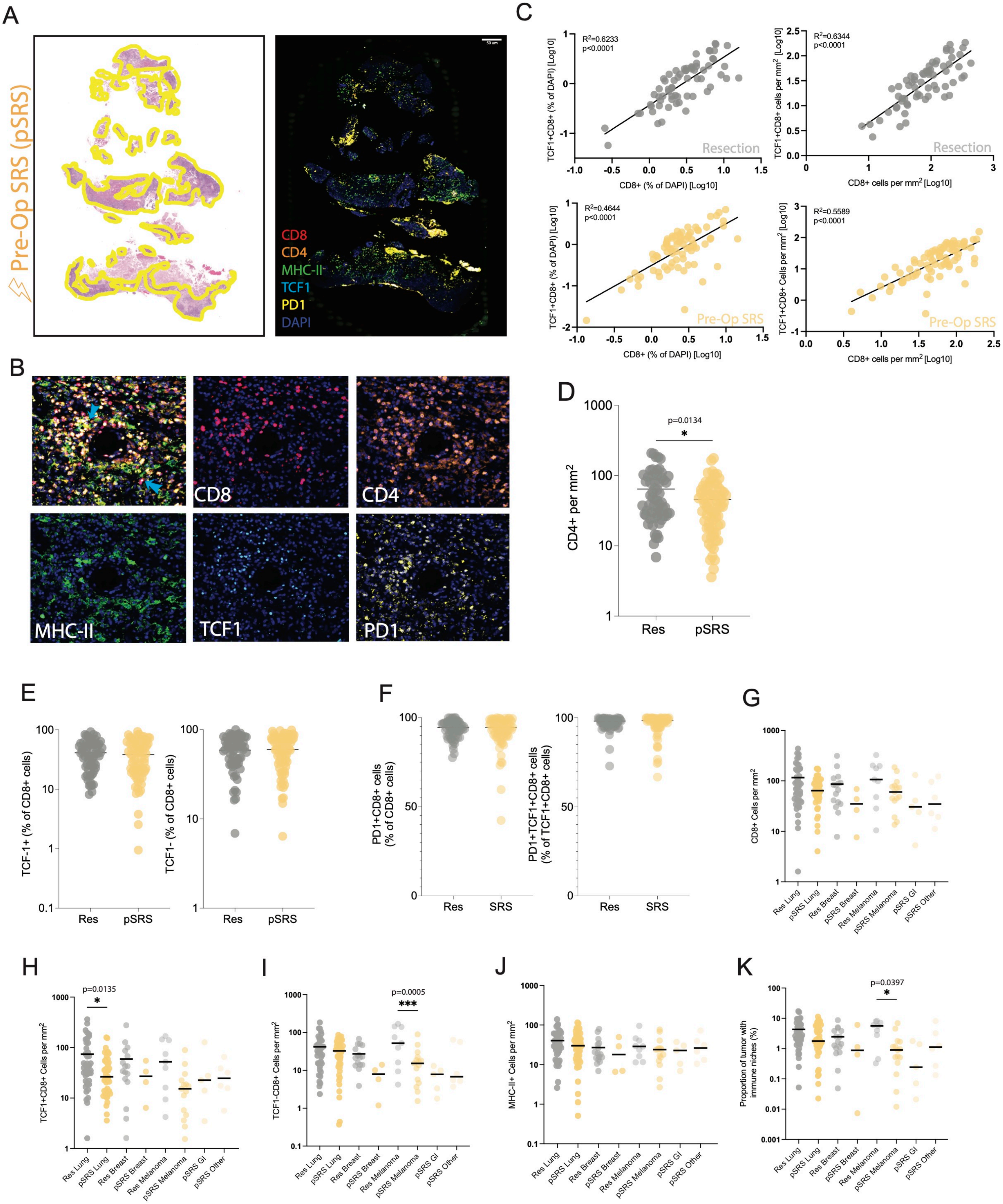
A
Pre-Op SRS (pSRS)
50 um
CD8
CD4
MHC-II
TCF1
PD1
DAPI
B
CD8
CD4
MHC-II
TCF1
PD1
C
R^2=0.6233
p<0.0001
TCF1+CD8+ (% of DAPI) [Log10]
CD8+ (% of DAPI) [Log10]
Resection
R^2=0.6344
p<0.0001
TCF1+CD8+ cells per mm^2 [Log10]
CD8+ cells per mm^2 [Log10]
Resection
R^2=0.4644
p<0.0001
TCF1+CD8+ (% of DAPI) [Log10]
CD8+ (% of DAPI) [Log10]
Pre-Op SRS
R^2=0.5589
p<0.0001
TCF1+CD8+ Cells per mm^2 [Log10]
CD8+ cells per mm^2 [Log10]
Pre-Op SRS
D
p=0.0134
*
CD4+ per mm^2
Res
pSRS
E
TCF-1+ (% of CD8+ cells)
TCF1- (% of CD8+ cells)
Res
pSRS
F
PD1+CD8+ cells (% of CD8+ cells)
PD1+TCF1+CD8+ cells (% of TCF1+CD8+ cells)
Res
SRS
G
CD8+ Cells per mm^2
Res Lung
pSRS Lung
Res Breast
pSRS Breast
Res Melanoma
pSRS Melanoma
pSRS GI
pSRS Other
H
p=0.0135
*
TCF1+CD8+ Cells per mm^2
I
p=0.0005
***
TCF1-CD8+ Cells per mm^2
J
MHC-II+ Cells per mm^2
K
p=0.0397
*
Proportion of tumor with immune niches (%)

Supplementary Figure 3. **A)** Whole slide H&E and immunofluorescence image of pSRS BrM. **B)** Composite and individual stain immunofluorescence images of pSRS BrM. **C)** Correlation plots of CD8 T cell frequency (% of total cells) vs. TCF1+ CD8 cell frequency (left) or CD8 cells per mm^2 vs. TCF1+ CD8 cell per mm^2 (right) for Res (top) and pSRS BrM (bottom). **D)** Quantification of CD4 per mm^2 in Res vs. pSRS BrM, **E)** Quantification of %TCF1+ and %TCF1- of CD8 cells in Res vs. pSRS BrM, **F)** Quantification of %PD1+ CD8 of CD8 cells and PD1+ TCF1+ CD8 of TCF1+ CD8 cells in Res or pSRS. For D-F, *:$p < 0.05$ as calculated by two-sided unpaired Mann–Whitney U test. **G-K)** Comparison of immune infiltrate across tumor histology by cell type for both Res and pSRS BrM: **G)** Quantification of CD8 cells per mm^2, **H)** TCF1+ CD8 cells per mm^2, **I)** TCF1- CD8 cells per mm^2, **J)** MHC-II+ per mm^2, **K)** Proportion of tumor with immune niches. In G-K, *:$p < 0.05$, ***:$p < 0.0005$, as calculated by ordinary one-way ANOVA, exact p-values shown in the figure. C-F) n=67 for Res and n=76 for pSRS for each plot; G-K) for Res n=42 lung, n=16 breast, n=9 melanoma, and for pSRS n=46 lung, n=4 breast, n=15 melanoma, n=5 gastrointestinal (GI), n=6 other. Source data are provided as a Source Data file.

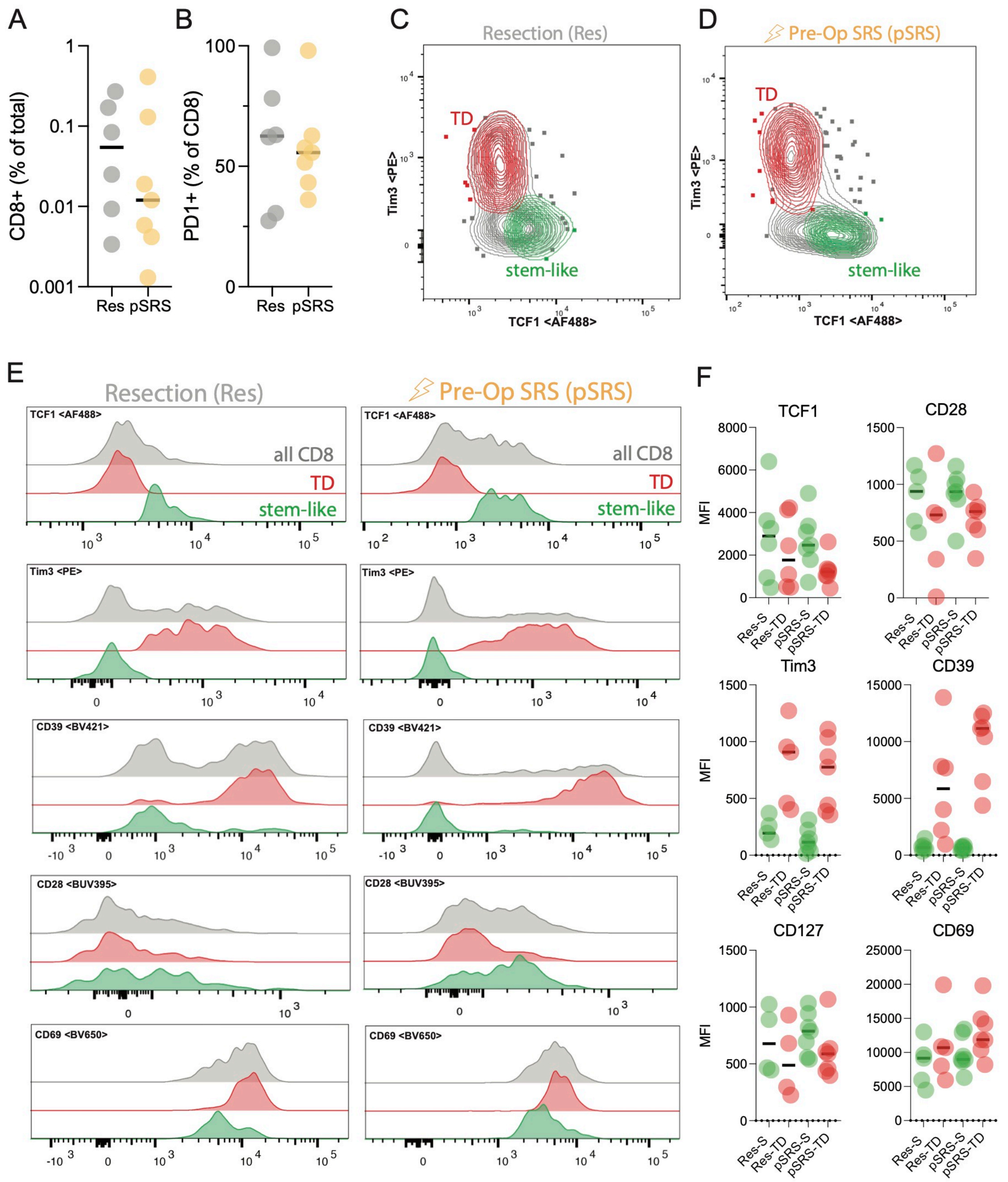
A
CD8+ (% of total)
Res pSRS
B
PD1+ (% of CD8)
Res pSRS
C
Resection (Res)
TD
stem-like
Tim3 <PE>
TCF1 <AF488>
D
Pre-Op SRS (pSRS)
TD
stem-like
Tim3 <PE>
TCF1 <AF488>
E
Resection (Res)
Pre-Op SRS (pSRS)
TCF1 <AF488>
all CD8
TD
stem-like
Tim3 <PE>
CD39 <BV421>
CD28 <BUV395>
CD69 <BV650>
F
TCF1
CD28
Tim3
CD39
CD127
CD69
MFI
Res-S
Res-TD
pSRS-S
pSRS-TD

Supplementary Figure 4. A) Frequency of CD8 T cells as measured by flow cytometry; n=6 for Res, n=7 for pSRS. **B)** Proportion of PD1+ cells of total CD8 T cells as measured by flow cytometry; n=6 for Res, n=7 for pSRS. **C-D)** Representative flow plots of stem-like and terminally differentiated effector-like T cells from **C)** Res and **D)** pSRS BrM. **E)** Representative histograms showing the MFI of multiple stem-like and TD T cell markers. **F)** Summary plots for MFI of various markers for stem-like and TD T cells by treatment condition. For TCF1, n=6 Res, n=7 pSRS; for CD28, n=5 Res, n=7 pSRS; for Tim3, n=5 Res, n=7 pSRS; for CD39, n=6 Res, n=7 pSRS; for CD127, n=4 Res, n=7 pSRS; for CD69, n=5 Res, n=7 pSRS. Source data are provided as a Source Data file.

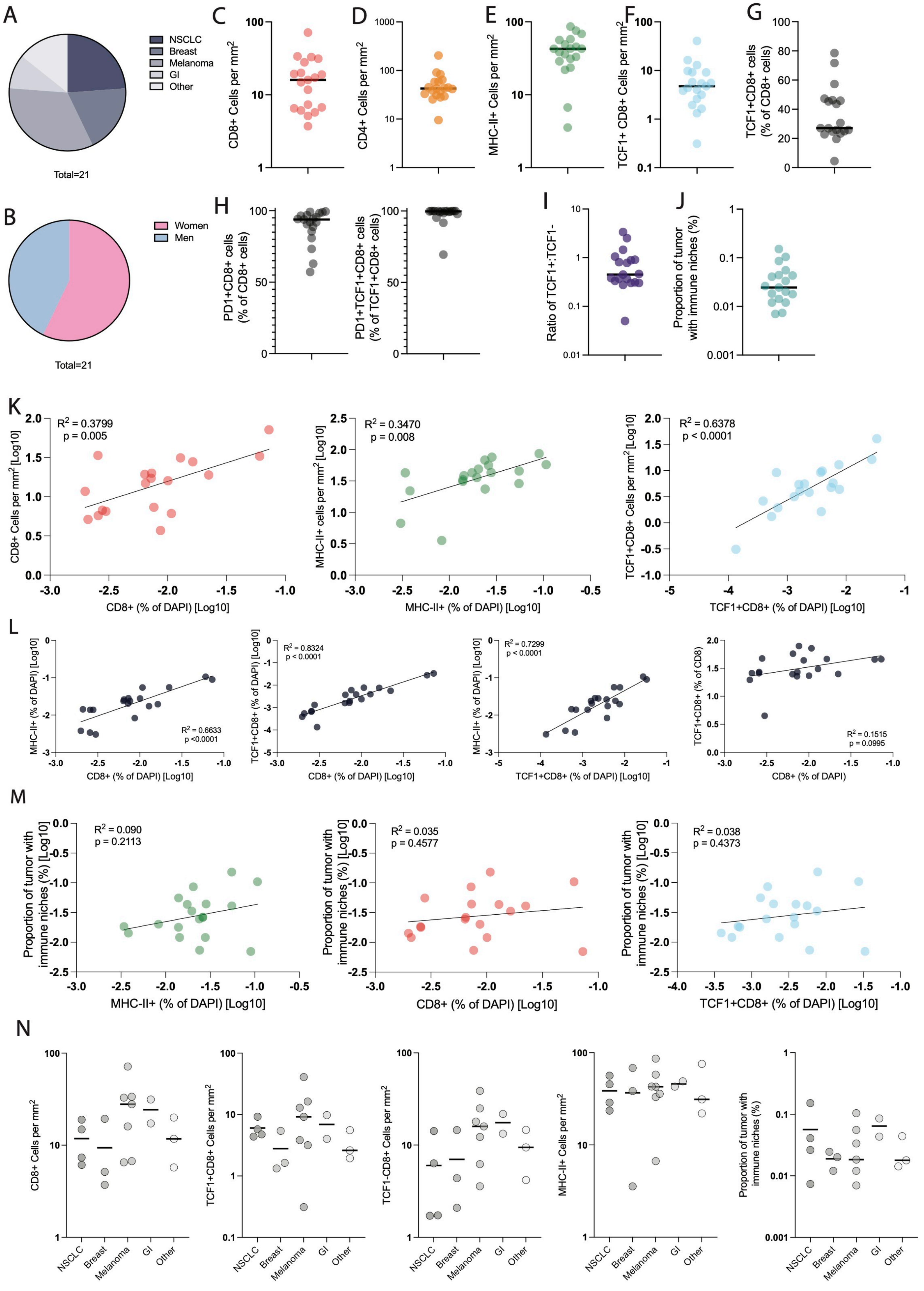

A
NSCLC
Breast
Melanoma
GI
Other
Total=21
B
Women
Men
Total=21
C
CD8+ Cells per mm2
D
CD4+ Cells per mm2
E
MHC-II+ Cells per mm2
F
TCF1+ CD8+ Cells per mm2
G
TCF1+CD8+ cells (% of CD8+ cells)
H
PD1+CD8+ cells (% of CD8+ cells)
PD1+TCF1+CD8+ cells (% of TCF1+CD8+ cells)
I
Ratio of TCF1+:TCF1-
J
Proportion of tumor with immune niches (%)
K
R2 = 0.3799
p = 0.005
CD8+ Cells per mm2 [Log10]
CD8+ (% of DAPI) [Log10]
R2 = 0.3470
p = 0.008
MHC-II+ cells per mm2 [Log10]
MHC-II+ (% of DAPI) [Log10]
R2 = 0.6378
p < 0.0001
TCF1+CD8+ Cells per mm2 [Log10]
TCF1+CD8+ (% of DAPI) [Log10]
L
MHC-II+ (% of DAPI) [Log10]
CD8+ (% of DAPI) [Log10]
R2 = 0.6633
p <0.0001
TCF1+CD8+ (% of DAPI) [Log10]
R2 = 0.8324
p < 0.0001
CD8+ (% of DAPI) [Log10]
MHC-II+ (% of DAPI) [Log10]
R2 = 0.7299
p < 0.0001
TCF1+CD8+ (% of DAPI) [Log10]
TCF1+CD8+ (% of CD8)
R2 = 0.1515
p = 0.0995
CD8+ (% of DAPI)
M
Proportion of tumor with immune niches (%) [Log10]
R2 = 0.090
p = 0.2113
MHC-II+ (% of DAPI) [Log10]
R2 = 0.035
p = 0.4577
CD8+ (% of DAPI) [Log10]
R2 = 0.038
p = 0.4373
TCF1+CD8+ (% of DAPI) [Log10]
N
CD8+ Cells per mm2
TCF1+CD8+ Cells per mm2
TCF1-CD8+ Cells per mm2
MHC-II+ Cells per mm2
Proportion of tumor with immune niches (%)
NSCLC
Breast
Melanoma
GI
Other

Supplementary Figure 5. A) Distribution of BrM histologies on prospective preoperative SRS clinical trial. **B)** Sex distribution of patients on trial. **C-J)** Quantitation of **C)** CD8 cells per mm^2, **D)** CD4 per mm^2, **E)** MHC-II+ cells per mm^2, **F)** TCF1+ CD8 cells per mm^2, **G)** Frequency TCF1+ CD8 cells of CD8 cells, **H)** frequency of PD1+ CD8 of CD8 cells and PD1+ TCF1+ CD8 of TCF1+ CD8 cells, **I)** Ratio of TCF1+ to TCF1- CD8 cells, and **J)** Proportion of BrM with immune niche. **K)** Correlation plots of CD8 cell frequency vs. CD8 cell per mm^2, MHC-II+ frequency vs. MHC-II+ cells per mm^2, and TCF1+ CD8 cell frequency vs. TCF1+ CD8+ cells per mm^2 for methodological validation. **L)** Multiple correlation plots of CD8 T cells subsets and MHC-II+ cells. **M)** Correlation plots of MHC-II+ frequency, CD8 cell frequency, TCF1+ CD8 frequency vs proportion of tumor with immune niches. **N)** Comparison of immune infiltrate across tumor histology by cell type and proportion of tumor with immune niche (one value not plotted due to log(0) undefined). For C-M, n=19; for N, n=4 NSCLC, n=3 breast, n=7 melanoma, n=2 gastrointestinal (GI), n=3 other. Source data are provided as a Source Data file.

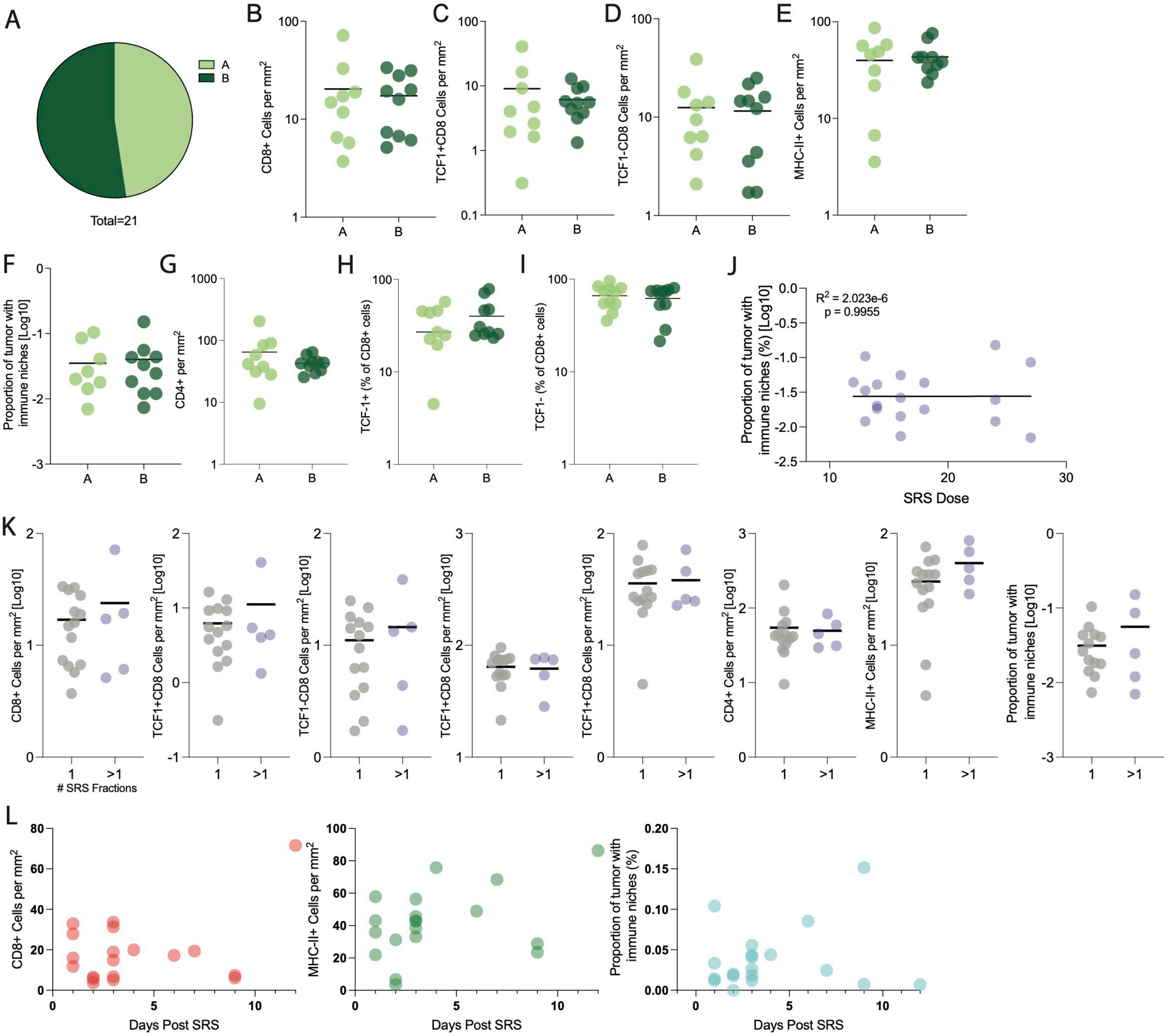
A
A
B
Total=21
B
CD8+ Cells per mm2
C
TCF1+CD8 Cells per mm2
D
TCF1-CD8 Cells per mm2
E
MHC-II+ Cells per mm2
F
Proportion of tumor with immune niches [Log10]
G
CD4+ per mm2
H
TCF-1+ (% of CD8+ cells)
I
TCF1- (% of CD8+ cells)
J
R2 = 2.023e-6
p = 0.9955
Proportion of tumor with immune niches (%) [Log10]
SRS Dose
K
CD8+ Cells per mm2 [Log10]
SRS Fractions
TCF1+CD8 Cells per mm2 [Log10]
TCF1-CD8 Cells per mm2 [Log10]
TCF1+CD8 Cells per mm2 [Log10]
TCF1+CD8 Cells per mm2 [Log10]
CD4+ Cells per mm2 [Log10]
MHC-II+ Cells per mm2 [Log10]
Proportion of tumor with immune niches [Log10]
L
CD8+ Cells per mm2
Days Post SRS
MHC-II+ Cells per mm2
Days Post SRS
Proportion of tumor with immune niches (%)
Days Post SRS

Supplementary Figure 6. A) Breakdown of enrollment of patients on the low dose dexamethasone (A) and the high dose (B) arms of the prospective pre-operative SRS trial. **B-I)** Comparison of quantification of immunofluorescence staining for immune cell infiltration between Arm A and Arm B; n=9 for A and n=10 for B. **B)** CD8 cells per mm^2, **C)** TCF1+ CD8 per mm^2, **D)** TCF1- CD8 per mm^2, **E)** MHC-II+ per mm^2, **F)** proportion of tumor with immune **G)** CD4 cells per mm^2 (one value not plotted due to log(0) undefined), **H)** TCF1+ (% of CD8+ cells), **I)** TCF1- (% of CD8+ cells). **J)** Correlation plot of pSRS dose and proportion of tumor with immune niche (n=19, one value not plotted due to log(0) undefined). **K)** Quantification of immune cell infiltrates divided by 1 vs ≥1 fraction of SRS (n=14 for 1 and n=5 for >1). **L**) Distribution of immune cell infiltrate quantification by days from pSRS to surgery (n=19 for each plot). Source data are provided as a Source Data file.

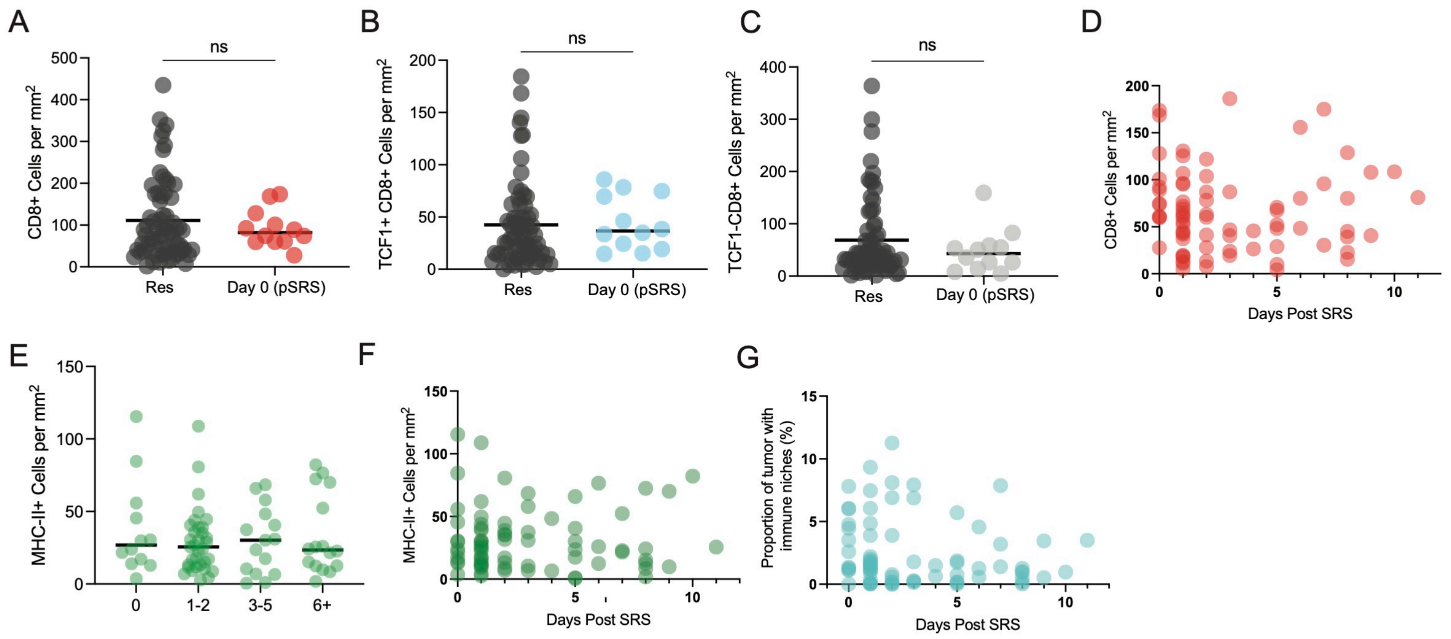

A
CD8+ Cells per mm2
ns
Res
Day 0 (pSRS)
B
TCF1+ CD8+ Cells per mm2
ns
Res
Day 0 (pSRS)
C
TCF1-CD8+ Cells per mm2
ns
Res
Day 0 (pSRS)
D
CD8+ Cells per mm2
Days Post SRS
E
MHC-II+ Cells per mm2
0
1-2
3-5
6+
F
MHC-II+ Cells per mm2
Days Post SRS
G
Proportion of tumor with immune niches (%)
Days Post SRS

Supplementary Figure 7. A-C) Quantification of **A)** CD8 cells per mm^2, **B)** TCF1+ CD8 cells per mm^2, and **C)** TCF1- CD8 cells per mm^2 for Res and pSRS who underwent same day surgical resection (n=67 Res, and n=12 pSRS day 0) **D)** Quantification of CD8 cells per mm^2 vs. time from pSRS to tumor resection (n=76). **E)** Quantification of MHC-II+ cells per mm^2 at discrete time points (n=12, 33, 15, 16 for days post SRS 0, 1-2, 3-5 and 6+ respectively). **F)** Quantification of MHC-II+ cells per mm^2 vs. time from pSRS to tumor resection (n=76). **G)** Quantification of proportion of tumor with immune niches vs. time from pSRS to tumor resection (n=76). Source data are provided as a Source Data file.

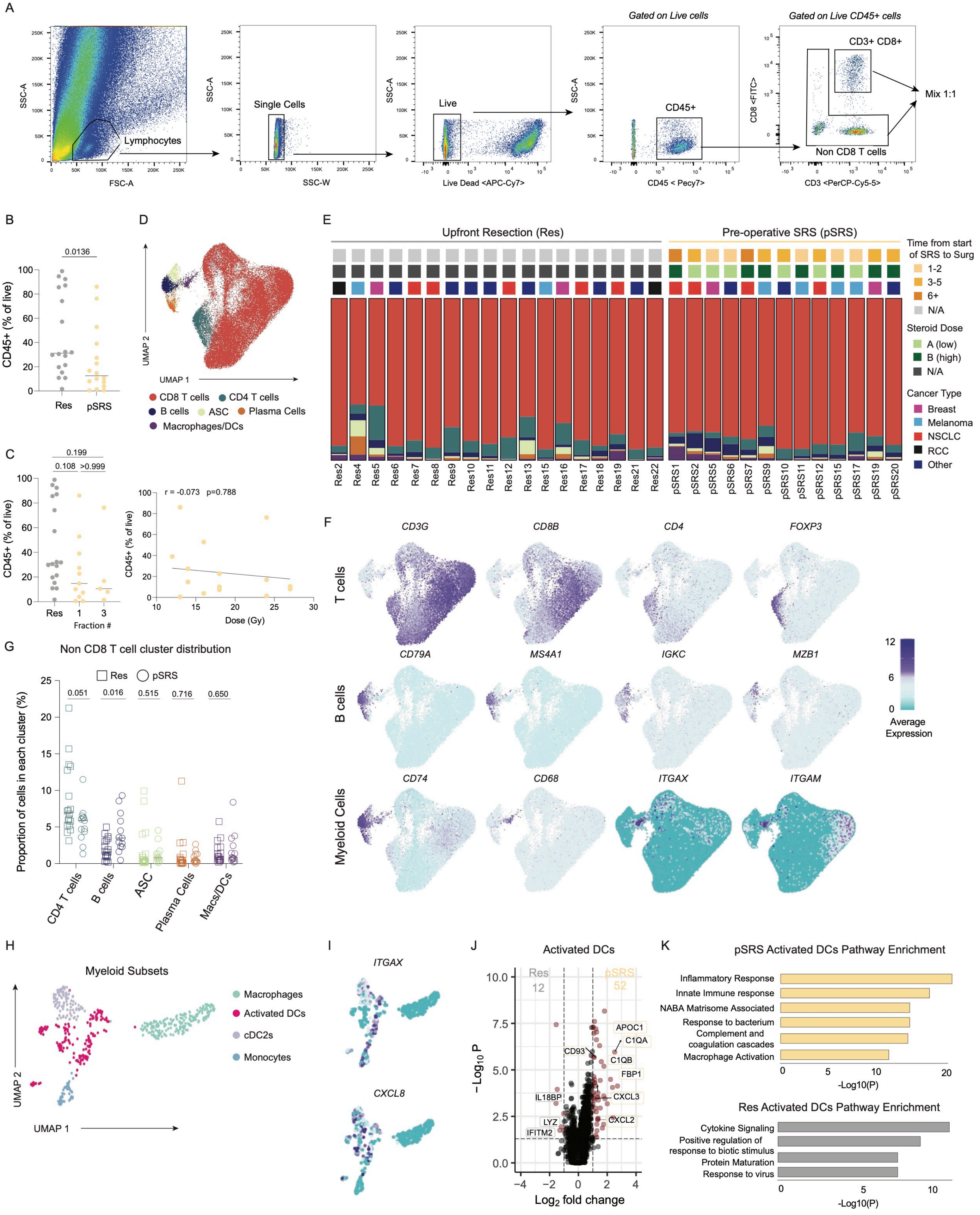
A
Gated on Live cells
Gated on Live CD45+ cells
Lymphocytes
Single Cells
Live
CD45+
CD3+ CD8+
Non CD8 T cells
Mix 1:1
SSC-A
FSC-A
SSC-W
Live Dead <APC-Cy7>
CD45 < Pecy7>
CD8 <FITC>
CD3 <PerCP-Cy5-5>
B
0.0136
CD45+ (% of live)
Res
pSRS
C
0.199
0.108
>0.999
Fraction #
r = -0.073 p=0.788
Dose (Gy)
D
UMAP 2
UMAP 1
CD8 T cells
CD4 T cells
B cells
ASC
Plasma Cells
Macrophages/DCs
E
Upfront Resection (Res)
Pre-operative SRS (pSRS)
Time from start of SRS to Surg
1-2
3-5
6+
N/A
Steroid Dose
A (low)
B (high)
N/A
Cancer Type
Breast
Melanoma
NSCLC
RCC
Other
Res2 Res4 Res5 Res6 Res7 Res8 Res9 Res10 Res11 Res12 Res13 Res15 Res16 Res17 Res18 Res19 Res21 Res22
pSRS1 pSRS2 pSRS5 pSRS6 pSRS7 pSRS9 pSRS10 pSRS11 pSRS12 pSRS15 pSRS17 pSRS19 pSRS20
F
T cells
CD3G
CD8B
CD4
FOXP3
B cells
CD79A
MS4A1
IGKC
MZB1
Myeloid Cells
CD74
CD68
ITGAX
ITGAM
Average Expression
G
Non CD8 T cell cluster distribution
Res
pSRS
0.051
0.016
0.515
0.716
0.650
Proportion of cells in each cluster (%)
CD4 T cells
B cells
ASC
Plasma Cells
Macs/DCs
H
Myeloid Subsets
Macrophages
Activated DCs
cDC2s
Monocytes
UMAP 2
UMAP 1
I
ITGAX
CXCL8
J
Activated DCs
Res 12
pSRS 52
APOC1
C1QA
CD93
C1QB
FBP1
IL18BP
CXCL3
CXCL2
LYZ
IFITM2
−Log10 P
Log2 fold change
K
pSRS Activated DCs Pathway Enrichment
Inflammatory Response
Innate Immune response
NABA Matrisome Associated
Response to bacterium
Complement and coagulation cascades
Macrophage Activation
-Log10(P)
Res Activated DCs Pathway Enrichment
Cytokine Signaling
Positive regulation of response to biotic stimulus
Protein Maturation
Response to virus
-Log10(P)

Supplementary Figure 8. **A)** Sorting layout for CD45+ and CD8 T cells from Res (n=13) or pSRS (n=18) BrM for single cell RNA-seq analysis. **B)** Frequency of live CD45+ cells from all patients on the day of sort analyzed by flow cytometry (n=34). Medians are shown and statistical comparisons were performed using two-sided unpaired Mann Whitney U test. **C)** Frequency of live CD45+ cells from all patients, as measured by flow cytometry. pSRS patients are split according to the pSRS fraction number they received (left). Medians are shown and statistical comparisons were performed using Kruskall Wallis test with Dunn's multiple comparisons tests. Right panel shows spearman correlation of the relative frequency of CD45+ cells from pSRS patients correlated with the pSRS dosage (Gy) received. P value is shown (n=18 Res, n=11 pSRS 1 fraction, n=5 pSRS 3 fractions). **D)** Single cell RNAseq UMAP projection of sorted CD45+ and CD8 T cells from patients with BrM (n=31). **E)** Relative distribution of clusters for each patient. Colored squares highlight the specific number of days from pSRS to surgery, steroid dose administered, and histology for each BrM sequenced. **F)** UMAP projections of normalized expression of selected genes defining each cluster. **G)** Relative frequency of non CD8 T cell clusters for each patient within Res and pSRS groups. Medians are shown and statistical comparisons were performed using two-sided unpaired Mann Whitney U test between Res and pSRS groups across populations (n=18 Res, n=13 pSRS). **H)** UMAP projection of sub-clustered myeloid populations and defined based on differential gene expression analysis. **I)** Normalized gene expression of selected genes upregulated in activated DCs. **J)** Transcriptional comparison of activated DCs between Res and pSRS groups. Volcano plot shows fold change vs -log(p-value) for each gene. Numbers at the top of each volcano plot show the number of genes differentially expressed between groups. **K)** Metascape pathway enrichment analysis in activated DCs from pSRS and Res BrM. Top five significant pathways are shown for each group. Source data are available on the NCBI Gene Expression Omnibus (GEO) database.

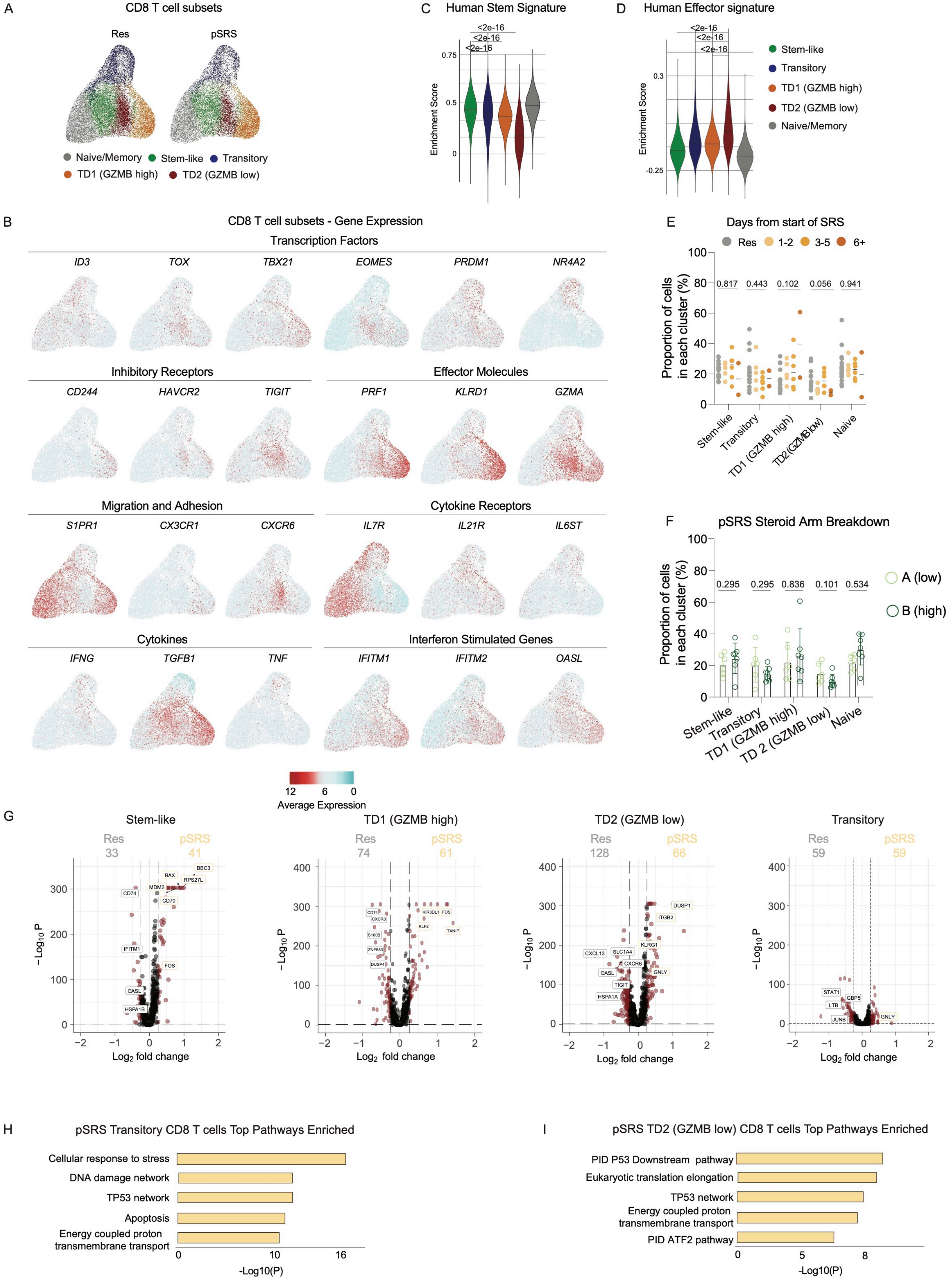
A
CD8 T cell subsets
Res
pSRS
Naive/Memory
Stem-like
Transitory
TD1 (GZMB high)
TD2 (GZMB low)
C
Human Stem Signature
<2e-16
<2e-16
<2e-16
0.75
0.5
0
Enrichment Score
D
Human Effector signature
<2e-16
<2e-16
<2e-16
0.3
-0.25
Enrichment Score
Stem-like
Transitory
TD1 (GZMB high)
TD2 (GZMB low)
Naive/Memory
B
CD8 T cell subsets - Gene Expression
Transcription Factors
ID3
TOX
TBX21
EOMES
PRDM1
NR4A2
Inhibitory Receptors
CD244
HAVCR2
TIGIT
Effector Molecules
PRF1
KLRD1
GZMA
Migration and Adhesion
S1PR1
CX3CR1
CXCR6
Cytokine Receptors
IL7R
IL21R
IL6ST
Cytokines
IFNG
TGFB1
TNF
Interferon Stimulated Genes
IFITM1
IFITM2
OASL
12
6
0
Average Expression
E
Days from start of SRS
Res
1-2
3-5
6+
Proportion of cells in each cluster (%)
0.817
0.443
0.102
0.056
0.941
Stem-like
Transitory
TD1 (GZMB high)
TD2 (GZMB low)
Naive
F
pSRS Steroid Arm Breakdown
A (low)
B (high)
Proportion of cells in each cluster (%)
0.295
0.295
0.836
0.101
0.534
Stem-like
Transitory
TD1 (GZMB high)
TD 2 (GZMB low)
Naive
G
Stem-like
Res 33
pSRS 41
BBC3
BAX
RPS27L
MDM2
CD74
CD70
IFITM1
FOS
OASL
HSPA1B
TD1 (GZMB high)
Res 74
pSRS 61
CD74
CXCR3
KIR3DL1
FOS
KLF2
TXNIP
S100B
ZNF683
DUSP4
TD2 (GZMB low)
Res 128
pSRS 66
DUSP1
ITGB2
KLRG1
CXCL13
SLC1A4
CXCR6
OASL
GNLY
TIGIT
HSPA1A
Transitory
Res 59
pSRS 59
STAT1
GBP5
LTB
JUNB
GNLY
−Log10 P
Log2 fold change
H
pSRS Transitory CD8 T cells Top Pathways Enriched
Cellular response to stress
DNA damage network
TP53 network
Apoptosis
Energy coupled proton transmembrane transport
0
10
16
-Log10(P)
I
pSRS TD2 (GZMB low) CD8 T cells Top Pathways Enriched
PID P53 Downstream pathway
Eukaryotic translation elongation
TP53 network
Energy coupled proton transmembrane transport
PID ATF2 pathway
0
5
8
-Log10(P)

Supplementary Figure 9. A) Single cell RNAseq UMAP projection of sub-clustered CD8 T cells in BrM for both Res and pSRS groups. **B)** Normalized gene expression of selected genes across CD8 T cell clusters with key transcription factors, as well as surface molecules and receptors that define CD8 T cell subsets. **C-D)** Gene set enrichment VISION analysis using the C) Human Stem-like and D) Human Effector/Terminally differentiated CD8 T cell signature. Signature enrichment score is represented as violin plots with the mean for each cluster shown and were analyzed by Kruskal-Wallis test with Benjamini-Hochberg adjustment for multiple comparisons, P values are shown. **E)** Relative frequency of CD8 T cell clusters in Res and pSRS tumors. pSRS patients were split based on the number of days from start of SRS to the date of surgery. Median is represented and CD8 T cell population frequencies were analyzed by Kruskal-Wallis test with Dunn's multiple comparisons tests , P values are shown (n=18 Res, n=5 pSRS days 1-2, n=6 pSRS days 3-5, n=2 pSRS days 6+). **F)** Relative frequency of CD8 T cell clusters in pSRS tumors split based on the dose of dexamethasone received prior to tumor resection. Mean +/- s.d. is represented and CD8 T cell population frequencies were analyzed by two-sided unpaired Mann–Whitney U test between Res and pSRS conditions, P values are shown (n=6 pSRS Arm A, n=7 pSRS Arm B). **G)** Transcriptional comparison of CD8 T cell clusters between Res and pSRS groups. Volcano plot shows fold change vs -log(p-value) for each gene. Numbers at the top of each volcano plot show the number of genes differentially expressed between groups. **H-I)** Metascape pathway enrichment analysis in CD8 T cell subsets from pSRS tumors. Top five significant pathways are shown for **H)** Transitory CD8 T cells and **I)** TD2 GZMB low CD8 T cells. Source data are available on the NCBI Gene Expression Omnibus (GEO) database.

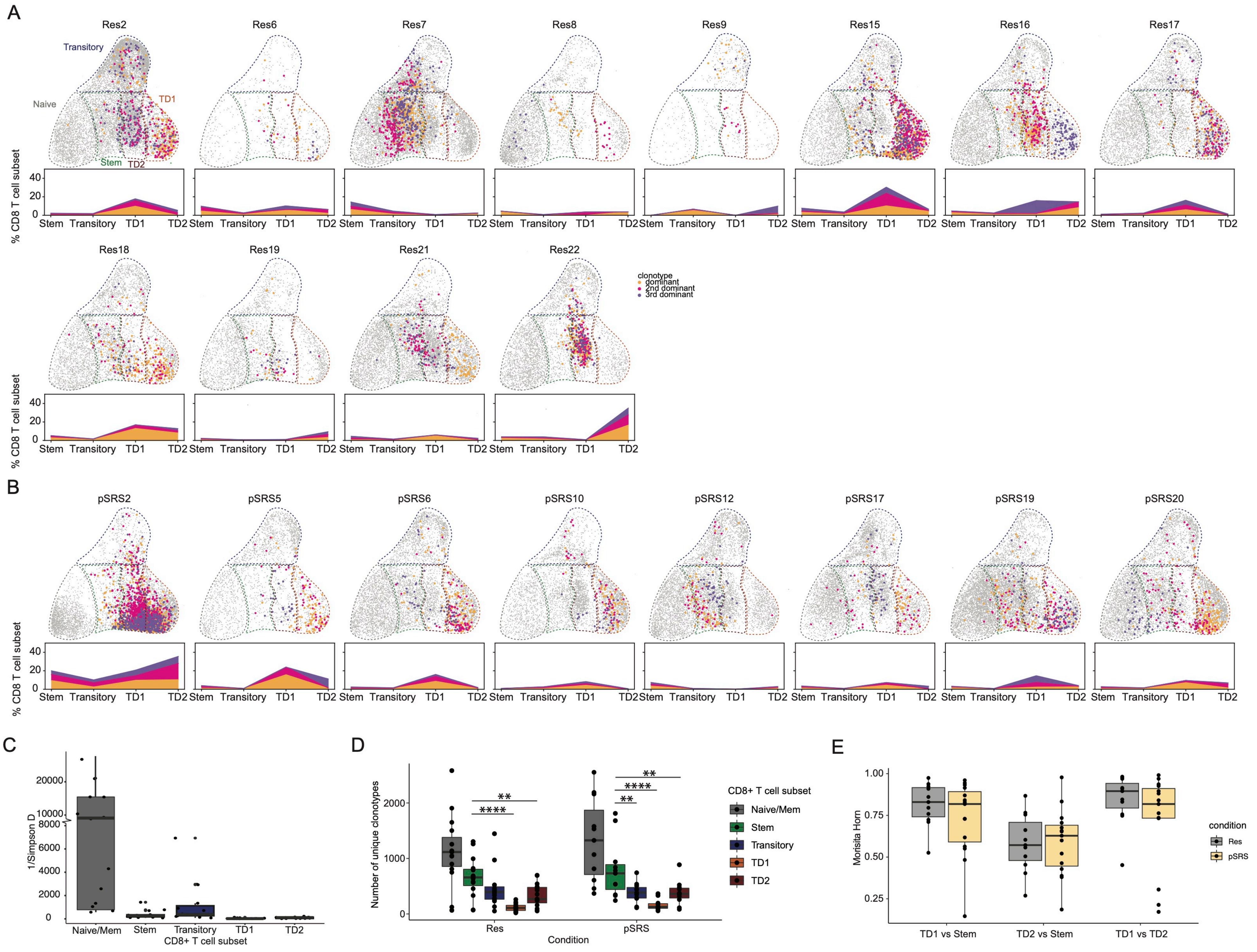
A
Res2
Res6
Res7
Res8
Res9
Res15
Res16
Res17
Transitory
Naive
TD1
Stem
TD2
% CD8 T cell subset
40
20
0
Stem
Transitory
TD1
TD2
Res18
Res19
Res21
Res22
clonotype
dominant
2nd dominant
3rd dominant
B
pSRS2
pSRS5
pSRS6
pSRS10
pSRS12
pSRS17
pSRS19
pSRS20
C
1/Simpson D
20000
10000
8000
6000
4000
2000
0
Naive/Mem
Stem
Transitory
TD1
TD2
CD8+ T cell subset
D
Number of unique clonotypes
2000
1000
0
**
****
**
****
**
Res
pSRS
Condition
CD8+ T cell subset
Naive/Mem
Stem
Transitory
TD1
TD2
E
Morisita Horn
1.00
0.75
0.50
0.25
TD1 vs Stem
TD2 vs Stem
TD1 vs TD2
condition
Res
pSRS

Supplementary Figure 10. A-B) Top 3 clonotypes for remaining patients (**A)** Res or **B)** pSRS), superimposed on the UMAP of the previously described CD8 T cell subsets (stem-like, transitory, TD1, and TD2), with percentage of all cells quantified per BrM below. **C)** Quantification of TCR diversity, demonstrating greatest diversity in the naïve/memory cluster (n=15 in each group). **D)** Quantification of the number of unique clonotypes per CD8 T cell subset, which progressively decreases from stem-like to the effector clusters. For Res **:$p=0.0056$ and ****:$p < 0.0001$, for pSRS stem vs. trans **:$p=0.0068$, stem vs. TD2 **:$p=0.0066$, and ****:$p < 0.0001$ as calculated by ordinary one-way ANOVA. **E)** Quantification of TCR similarity (Morisita-Horn index) between stem/TD1, stem/TD2, and TD1/TD2. For D-E, n=15 Res and n=11 pSRS. Source data are available on the NCBI Gene Expression Omnibus (GEO) database.

Supplementary Table 1 – Entire Study Cohort by Arm

Covariate	Statistics	Level	Study Arm		P-value*
			A N=13	B N=13	
ECOG	N (Col %)	<2	11 (84.62)	7 (53.85)	0.202
	N (Col %)	>=2	2 (15.38)	6 (46.15)	
Gender	N (Col %)	F	6 (46.15)	7 (53.85)	0.695
	N (Col %)	M	7 (53.85)	6 (46.15)	
Primary Site	N (Col %)	NSCLC	4 (30.77)	3 (23.08)	0.915
	N (Col %)	Breast	1 (7.69)	3 (23.08)	
	N (Col %)	Melanoma	3 (23.08)	4 (30.77)	
	N (Col %)	RCC	1 (7.69)	0 (0)	
	N (Col %)	GI	1 (7.69)	1 (7.69)	
	N (Col %)	Other	3 (23.08)	2 (15.38)	
Previous IO therapy	N (Col %)	No	9 (69.23)	11 (84.62)	0.645
	N (Col %)	Yes	4 (30.77)	2 (15.38)	
Age	N		11	12	0.281
	Mean		62.8	58.01	
	Median		64.32	61.46	
	Min		49.18	34.51	
	Max		74.57	74.01	
	Std Dev		7.56	12.36	
WBC	N		13	13	0.254
	Mean		9.57	11.92	
	Median		8.7	12.4	
	Min		4.6	4.1	
	Max		18.8	23.3	
	Std Dev		4.05	5.99	
ANC	N		13	12	0.220
	Mean		6.85	9.34	
	Median		6.23	9.02	
	Min		2.21	1.43	
	Max		16.22	19.79	
	Std Dev		4.08	5.7	

Supplementary Table 1 – Entire Study Cohort by Arm

Covariate	Statistics	Level	Study Arm A N=13	Study Arm B N=13	P-value*
Lymphocytes	N		13	12	0.735
	Mean		1.89	1.64	
	Median		1.85	0.77	
	Min		0.6	0.34	
	Max		6.42	7.31	
	Std Dev		1.51	2.04	

* The p-value is calculated by ANOVA for numerical covariates; and chi-square test or Fisher's exact for categorical covariates, where appropriate. All tests are two-sided.

Supplementary Table 2 – Res Descriptive Statistics

Variable	Level	N (%) = 67
ECOG	<2	55 (82.1)
	>=2	12 (17.9)
Gender	Male	25 (37.3)
	Female	42 (62.7)
Age	Mean	58.61
	Median	58.00
	Minimum	32.00
	Maximum	83.00
	Std Dev	11.62
	Missing	0
Primary Site	Lung	42 (62.7)
	Breast	16 (23.9)
	Melanoma	9 (13.4)
Dexamethasone Dose (mg/day)	4	2 (5.0)
	8	4 (10.0)
	10	1 (2.5)
	12	7 (17.5)
	16	18 (45.0)
	24	6 (15.0)
	32	1 (2.5)
	40	1 (2.5)
	Missing	27
Post-op SRS dose (Gy)	13	1 (1.5)
	15	21 (31.8)
	16.5	1 (1.5)
	18	23 (34.8)
	20	1 (1.5)
	21	6 (9.1)
	24	1 (1.5)
	25	1 (1.5)
	30	10 (15.2)
	35	1 (1.5)
	Missing	1

Supplementary Table 2 – Res Descriptive Statistics

Variable	Level	N (%) = 67
Fractions	1	51 (76.1)
	3	3 (4.5)
	4	1 (1.5)
	5	12 (17.9)
BrM Volume (cm^3)	Mean	13.23
	Median	8.90
	Minimum	1.50
	Maximum	39.50
	Std Dev	11.45
	Missing	33
ANC	Mean	8.12
	Median	8.35
	Minimum	2.57
	Maximum	14.26
	Std Dev	2.97
	Missing	36
ALC	Mean	1.19
	Median	1.02
	Minimum	0.26
	Maximum	3.17
	Std Dev	0.76
	Missing	37

Supplementary Table 3 – Univariate Associations for Res and Immune Markers

Univariate Association with CD8 cells per mm2

			CD8/mm2	
Variable	Level	N	Median	Kruskal-Wallis P-value
ECOG	<2	55	87.48	0.503
	>=2	12	78.74	
Gender	Male	25	87.48	0.866
	Female	42	82.26	
Primary Site	Lung	42	81.85	0.506
	Breast	16	73.50	
	Melanoma	9	106.25	

		CD8/mm2	
Variable	N	Spearman CC	Spearman P-value
Age	67	0.032	0.797
Steroid Dose (mg/day)	40	0.220	0.173
Volume	34	-0.174	0.324

Univariate Association with TCF percentage of CD8

			TCF % of CD8	
Variable	Level	N	Median	Kruskal-Wallis P-value
ECOG	<2	55	39.31	0.719
	>=2	12	43.08	
Gender	Male	25	36.23	0.509
	Female	42	40.78	
Primary Site	Lung	42	36.36	0.198
	Breast	16	38.42	
	Melanoma	9	53.01	

		TCF % of CD8	
Variable	N	Spearman CC	Spearman P-value
Age	67	0.150	0.224
Steroid Dose (mg/day)	40	0.019	0.906
Volume	34	0.126	0.477

Univariate Association with Niche Proportion

			Niche Proportion	
Variable	Level	N	Median	Kruskal-Wallis P-value
ECOG	<2	55	2.63	0.794
	>=2	12	2.96	
Gender	Male	25	3.60	0.364
	Female	42	2.45	
Primary Site	Lung	42	2.68	0.134
	Breast	16	1.53	
	Melanoma	9	5.57	

		Niche Proportion	
Variable	N	Spearman CC	Spearman P-value
Age	67	0.243	0.113
Steroid Dose (mg/day)	40	0.132	0.416
Volume	34	-0.335	0.053

The p-value is calculated by Kruskal-Wallis test for categorical covariates and Spearman rank correlation test is used for numerical covariates. All tests are two-sided.

Supplementary Table 4 - pSRS patient/BrM characteristics

Variable	Level	N (%) = 76
ECOG	<2	59 (77.6)
	>=2	17 (22.4)
Gender	Male	40 (52.6)
	Female	36 (47.4)
Primary Site	Lung	46 (60.5)
	Breast	4 (5.3)
	Melanoma	15 (19.7)
	GI	5 (6.6)
	Other	6 (7.9)
Preop SRS dose (Gy)	12	1 (1.3)
	13	7 (9.2)
	13.5	3 (3.9)
	14	11 (14.5)
	14.5	2 (2.6)
	15	19 (25.0)
	16	13 (17.1)
	17	7 (9.2)
	18	7 (9.2)
	19	1 (1.3)
	21	1 (1.3)
	22	2 (2.6)
	27	1 (1.3)
	30	1 (1.3)
Dexamethasone Dose (mg/day)	0	1 (2.0)
	4	5 (10.0)
	6	2 (4.0)
	8	11 (22.0)
	12	11 (22.0)
	16	19 (38.0)
	24	1 (2.0)
	Missing	26
Preop SRS fractions	1	74 (97.4)
	3	1 (1.3)
	5	1 (1.3)

Supplementary Table 4 - pSRS patient/BrM characteristics

Variable	Level	N (%) = 76
Age	Mean	61.06
	Median	59.77
	Minimum	26.47
	Maximum	95.30
	Std Dev	12.13
	Missing	0.00
Days from pre-op SRS to surgery	Mean	3.03
	Median	2.00
	Minimum	0.00
	Maximum	11.00
	Std Dev	2.94
	Missing	0.00
ANC	Mean	10.62
	Median	9.10
	Minimum	1.90
	Maximum	31.40
	Std Dev	6.79
	Missing	43.00
ALC	Mean	1.44
	Median	1.10
	Minimum	0.40
	Maximum	5.20
	Std Dev	1.08
	Missing	43.00

Supplementary Table 5 Descriptive Statistics for Res vs pSRS

Covariate	Statistics	Level	Group		P-value*
			Res N=67	pSRS N=76	
ECOG	N (Col %)	<2	55 (82.09)	59 (77.63)	0.508
	N (Col %)	>=2	12 (17.91)	17 (22.37)	
Sex	N (Col %)	Male	25 (37.31)	40 (52.63)	0.066
	N (Col %)	Female	42 (62.69)	36 (47.37)	
Age	N		67	76	0.221
	Mean		58.61	61.06	
	Median		58	59.77	
	Min		32	26.47	
	Max		83	95.3	
	Std Dev		11.62	12.13	
Dexamethasone Dose (mg/day)	N		40	50	**0.002**
	Mean		15.95	11.6	
	Median		16	12	
	Min		4	0	
	Max		40	24	
	Std Dev		6.99	4.85	
Volume	N		34	76	0.243
	Mean		13.23	11.03	
	Median		8.9	9.61	
	Min		1.5	1.3	
	Max		39.5	46.77	
	Std Dev		11.45	7.81	

* The p-value is calculated by ANOVA for numerical covariates; and chi-square test or Fisher's exact for categorical covariates, where appropriate. All tests are two-sided.

Supplementary Table 6

Sample Type	Source	Relevant Figures
Res (upfront resection) – IF	Emory brain tumor bank	2, 3, Supp 1, 2, 3
Res – Flow, scRNA/VDJ seq	Emory tissue collection	2, 4, 5, Supp 1, 8, 9, 10
pSRS – IF, Flow, scRNA/VDJ seq	Clinical Trial NCT04895592	3, 4, 5, Supp 4, 5, 6, 8, 9, 10
pSRS – IF	LCI tumor bank	3, Supp 3, 7

Supplementary Table 7 Antibodies

Target Antibody	Type	Clone	Concentration	Fluorophore	Vendor
CD8	Mouse IgG1, k	C8/144B	1:100	Opal 570	eBiosciences
CD4	Rabbit	EPR6855	1:300	Opal 690	Abcam
TCF1	Rabbit	C63D9	1:200	Opal 520	Cell Signalling
MHC-II	Mouse IgG2a, k	Tu39	1:75	Opal 620	BioLegend
PD1	Mouse IgG2a, k	EH33	1:100	Opal 480	Millipore/Sigma
Cytokeratin	Mouse IgG1, k	AE1/AE3	1:500	Opal 780	BioLegend
CD4	Mouse IgG2b, k	OKT4	1:100	BUV496	BioLegend
CD8	Mouse IgG1, k	RPA-T8	1:100	BUV661	BioLegend
PD1	Mouse IgG1, k	EH12.1	1:100	BUV737	BD Biosciences
CD39	Mouse IgG1, k	A1	1:100	BV421	BioLegend
CD45RA	Mouse IgG2b, k	HI100	1:100	BV510	BioLegend
CD3	Mouse IgG1, k	UCHT1	1:100	PerCP-Cy5.5	BioLegend
Tim3	Rat IgG2a	344823	1:50	PE	R&D Systems
CD28	Mouse IgG1, k	CD28.2	1:100	BUV395	Invitrogen
CD127	Mouse IgG1, k	A019D5	1:100	PE-Cy7	BioLegend
CCR7	Mouse IgG2a, k	G043H7	1:100	BV785	BioLegend
HLA-DR	Mouse IgG2a, k	L243	1:100	BV605	BioLegend
Tox	Rat IgG2a, k	TXR10	1:100	eFluor 660	Invitrogen
GranzymeB	Mouse IgG1, k	GB11	1:100	A700	BD Biosciences
TCF1	Rabbit	C63D9	1:100	AF488	Cell Signaling Technology
Ki67	Mouse IgG1, k	B56	1:50	BV711	BD Biosciences
CD69	Mouse IgG1, k	FN50	1:100	BV650	BioLegend
Foxp3	Mouse IgG1, k	206D	1:100	PE/Dazzle594	BioLegend

PROTOCOL TITLE: Preoperative radiosurgery for brain metastases planned for surgical resection: a two arm pilot study

WINSHIP PROTOCOL #: RAD5234-21

COORDINATING CENTER: Emory University

Principal Investigator(s):
Zachary Buchwald MD, PhD
Assistant Professor, Radiation Oncology
Emory University SOM
Telephone: 404-778-1790
zbuchwal@emory.edu

Co-Investigators:

Department of Radiation Oncology:

Hui-Kuo Shu MD PhD
Professor
Emory University SOM
hgshu@emory.edu

Kristin Higgins MD
Associate Professor
Emory University SOM
kristin.higgins@emory.edu

Jim Zhong MD
Assistant Professor
Emory University SOM
jim.zhong@emory.edu

Mohammad Khan MD PhD
Associate Professor
Emory University SOM
m.k.khan@emory.edu

Jeffrey Bradley MD
Professor
Emory University SOM
jeffrey.d.bradley@emory.edu

Department of Neurosurgery:

Jeffery Olson
Professor
Emory University SOM
jolson@emory.edu

Kimberly Hoang
Assistant Professor
Emory University SOM
kimberly.bojanowski.hoang@emory.edu

Edjah K. Nduom
Associate Professor
Emory University SOM
edjah.k.nduom@emory.edu

Translational Science:

Haydn Kissick PhD
Assistant Professor
Immunology/Microbiology
haydn.kissick@emory.edu

Carey Jansen B.S.
MD/PhD Student
Immunology/Microbiology
carey.jansen@emory.edu

Biostatistics:

Jeff Switchenko PhD, MS
Assistant Professor
Biostatistics and Bioinformatics
Rollins School of Public Health
jswitch@emory.edu

FUNDING SOURCE: NIH K12 Grant

INVESTIGATIONAL PRODUCT (IP): [*N/A*]
OTHER AGENT(S): Dexamethasone

IND # : N/A
☑ **Study Exempt from IND Requirements per 21 CFR 312.2(b).**

REVISION HISTORY

**Revision #**	**Version Date**	**Summary of Changes**
v2	3/1/21	Per CTRC request, updates made to the following: Objectives, Endpoints, arm allocation, DLT definition, statistical analysis plan
v3	4/15/21	Listed "Dexamethasone" above as "OTHER AGENT(S)." Removed references to "IND Sponsor."
v4	4/20/21	Section 4: Clarified dexamethasone dosing/billing.<br>Section 21: Removed paragraphs about multi-site participation as this is not applicable.
v5	6/1/21	Section 1.3: Schedule of Assessments:<br>Increased Screening window from 7 to 14 days.<br>Increased Study Visit 4 window from 3-22 days to 3-23 days.<br>Removed references to CRFs.<br>Section 9:<br>Changed "*Prior or suspected diagnosis of malignancy.*"<br>To: "*Prior pathologically confirmed or suspected extracranial diagnosis of malignancy*."

Table of Contents

1. Study Summary 3
1.1 Synopsis 3
1.2 Schema 4
1.3 Schedule of Assessments 4
2. Objectives (and Endpoints) 5
3. Background 5
3.1 Study Rationale 9
3.2 Clinical Experience 9
4. Study Intervention/Investigational Agent 10
5. Procedures Involved 10
5.1 Study Design 10
5.2 Dosing and Administration 11
5.3 Dose Modification ....
5.4 Study Procedures 11
5.5 Description of Study Procedures 12
6. Data and Specimen Banking 16
7. Sharing of Results with Participants 17
8. Study Timelines 17
8.1 Duration of therapy 13
8.2 Duration of follow-up 13
9. Inclusion and Exclusion Criteria 13
10. Local Number of Participants 14
11. Recruitment Methods 14
12. Withdrawal of Participants 15
13. Risks to Participants 19
14. Potential Benefits to Participants 15
15. Data Management and Confidentiality 16
15.1 Statistical consideration section: Biostatistician 20
15.2 Data/specimens: 21
16. Provisions to Monitor the Data to Ensure the Safety of Participants 22
17. Provisions to Protect the Privacy Interests of Participants 23
18. Economic Burden to Participants 23
19. Consent Process 23
20. Setting 25
21. Resources Available 25
22. References 27
APPENDIX APERFORMANCE STATUS CRITERIA 30
APPENDIX B Abbreviations and definition of terms 31

1. Study Summary

1.1 Synopsis

**Title:**	Preoperative Radiosurgery For Brain Metastases Planned For Surgical Resection, a two arm pilot study
**Study Description:**	This study will investigate the safety and feasibility of administering pre-operative radiosurgery followed by surgical resection at our institution. We will also investigate the impact of high vs low dose dexamethasone on intra-cranial T-cells following radiosurgery.
**Objectives:**	**Primary Objective**:<br>• To determine the safety of pre-operative SRS followed by surgery for brain metastasis<br>**Secondary Objectives:**<br>• To evaluate the immune niche in brain metastasis following SRS in the presence of low or high dose dexamethasone<br>• To evaluate the impact of pre-operative SRS on intracranial and survival outcomes
**Endpoints:**	**Primary Endpoint:**<br>• CTCAE grade 3 or greater toxicity at 4 months post-treatment<br><br>**Secondary Endpoint:**<br>• Density of immune niche in brain metastases<br>• Time to intracranial outcomes including local recurrence, anywhere brain failure<br>• Overall survival time.
**Primary Hypothesis**	The primary hypothesis of this pilot study is that the use of pre-operative SRS in the presence of low or high dose dexamethasone will be safe and well-tolerated.
**Secondary Hypothesis**	The patients receiving pre-operative SRS and low dose dexamethasone will have higher immune niche density in their brain metastasis
**Study Population:**	Patients with brain metastases
**Phase:**	Feasibility/Pilot
**Description of Sites/Facilities Enrolling Participants:**	Winship Cancer Institute of Emory University (Atlanta, GA).
**Description of Study Intervention:**	Stereotactic radiosurgery followed by resection of brain metastasis
**Study Duration:**	2 years

1.2 Schema

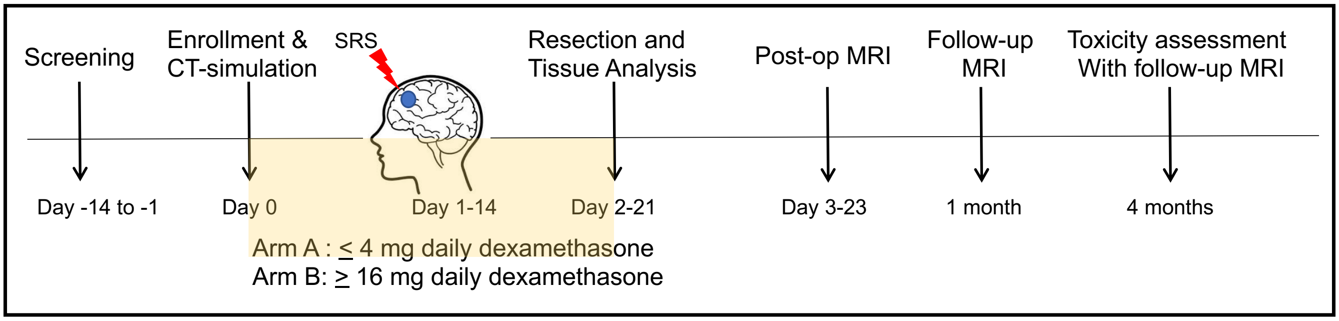

1.3 Schedule of Assessments

Procedures	**Screening** Day -14 to -1	**Enrollment/Baseline** Visit 1, Day 0	**Study Visit 2** Day 1-14	**Study Visit 3** Days 2-21	**Study Visit 4** Days 3-23	**Study Visit 5** Day 39 +/- 14 days	**Final Study Visit 6** Day 120 +/- 14 days
Informed consent	X						
Demographics	X						
Medical history	X						
Alternating enrollment on Arm A and B		X					
Administer SRS			X				
Perform Surgery				X			
Con. medication review	X					X	X
Physical exam (including height and weight)	X	X			X	X	X
Vital signs	X	X			X		
Height	X						
Weight	X	X		X		X	
Performance status	X	X		X	X	X	X
Hematology	X			X		X	X
serum chemistry	X			X		X	X
Pregnancy test	X						
AEs review and evaluation		X	X	X	X	X	X

**Procedures**	**Screening** Day -14 to -1	**Enrollment/Baseline** Visit 1, Day 0	**Study Visit 2** Day 1-14	**Study Visit 3** Days 2-21	**Study Visit 4** Days 3-23	**Study Visit 5** Day 39 +/- 14 days	**Final Study Visit 6** Day 120 +/- 14 days
Radiologic/Imaging assessment	X				X	X	X
Other assessments (e.g., flow/immunology assays on blood,)		X		X		X	X

2. Objectives (and Endpoints)

Describe the purpose, specific aims, or objectives and state the hypotheses to be tested.

OBJECTIVES	ENDPOINTS
Primary	
• To determine the safety of pre-operative SRS followed by surgery for brain metastasis	• <u>Safety</u>: CTCAE grade 3 or greater at 4 months post-treatment
Secondary	
• To evaluate the immune niche in brain metastasis following SRS in the presence of low or high dose dexamethasone<br>• To evaluate the impact of pre-operative SRS on survival outcomes	• Density of immune niche in brain metastases<br>• Time to intracranial outcomes including local recurrence, anywhere brain failure<br>• Overall survival time.

2.2 Background

Patients with solid cancers have a brain metastasis incidence of up to 30%, and these metastatic lesions are a significant source of morbidity and mortality. Lung, breast, and melanoma primaries are the most associated with brain metastasis development and account for 67%–80% brain metastasis cases. Stereotactic radiosurgery (SRS) has become the preferred therapy for patients with a limited number of brain metastases (defined as 1–4) based on multiple Phase III trials demonstrating significant reduction in the risk of neurocognitive and quality-of-life decline and no detriment in overall survival (OS) with the omission of whole brain radiotherapy (WBRT).[2-5] In the setting in which resection of brain metastasis is performed, resection alone has an expected 1–2 year local recurrence (LR) rate of 47%–59%[5-7]; hence radiotherapy (RT) as an adjunct to resection is generally recommended to reduce risk of cavity LR.

SRS can be administered pre-operatively or post-operatively. A group we have collaborated with, has previously demonstrated the feasibility of preoperative SRS given as a single fraction prior to

planned resection in a cohort of 47 patients.[8] They have also shown that preoperative SRS (in a cohort of 66 patients) has the potential to reduce the risk of radiation necrosis (RN) and leptomeningeal disease (LMD) recurrence with similar cavity LR rates compared with postoperative SRS.[9] Additionally, his group has demonstrated that preoperative SRS is not associated with increased risk of LMD compared with postoperative whole brain RT.[10] Most recently a published retrospective experience demonstrated continued excellent local control and radiation necrosis rates in an updated analysis with increased patient numbers and follow-up time.[11]

Despite the potential clinical benefit from SRS in the pre-operative setting, failure outside the radiation field (distant brain recurrence) is common, occurring in 30%-50% of patients at 12 months following SRS.[12-14] Whole-brain irradiation and/or immunotherapy are alternative strategies for patients who are at high risk for distant brain failure.[15,16] Moreover, while checkpoint blockade is standard for many advanced malignancies, several studies now show intracranial activity specifically in patients with BM.[17-19] **Therefore, given alternate and adjunctive management strategies, determining means to enhance the intra-cranial immune response is of great clinical importance.** Interestingly, higher numbers of CD8+ T-cells in primary and extra-cranial metastatic sites from different cancers including NSCLC and melanoma is associated with longer progression free (PFS), overall survival (OS), and response to immunotherapy.[20-23] However, CD8+ T cell infiltrate numbers alone in BMs specifically, have not always correlated with longer survival[24,25] suggesting that this one cell type may not fully capture the complexity and organization of the tumor microenvironment.[26,27] *Therefore, there remains a need for clinical interventions which enhance the intra-cranial anti-tumor response rates to checkpoint inhibitors.*

Recently, one of my mentors, Dr. Haydn Kissick, described an immune niche within primary renal cell carcinoma tumors consisting of antigen presenting cells (MHC-II$^+$) closely associated with a specific subset of T-cells known as TCF-1$^+$ stem-like CD8$^+$ T-cells. The density of MHC-II$^+$ cells correlated with longer PFS (**Figure 1**).[1] Unpublished data from this cohort indicates a strong correlation between the primary and metastatic site niche densities. Additionally, my other mentor, Dr. Rafi Ahmed, found that this stem-like subset of CD8$^+$ T-cells are particularly important for response to anti-PD-1/L1 therapy.[28] Additionally, following anti-PD-1 treatment these stem-like T-cells produce differentiated effector cells (Tim-3$^+$ TCF-1$^-$) capable of tumor killing.[28] Given the known immunogenicity of melanoma and efficacy of immune checkpoint blockade in this disease, **we hypothesize that the density of these immune niches will strongly correlate with brain recurrence in patients with melanoma BMs.** No previous study has evaluated the relationship between these immunological niches and intracranial BMs in melanoma patients. In preliminary experiments, we demonstrated the feasibility of identifying immune niches with immunofluorescence in 5 resected BM of different histologies including melanoma and lung adenocarcinoma collected from Emory patients. We see CD8$^+$ TCF-1$^+$ T-cell infiltrates adjacent to MHC-II$^+$ cells forming an immune niche as previously described[1] **(Figure 2)**. *__Further study is needed, however, within a larger and exclusively melanoma cohort to determine if niche density in BM is prognostic for intra-cranial outcomes, and whether density may be altered by immunosuppressive treatments like glucocorticoids or immunogenic treatments like radiation.__*

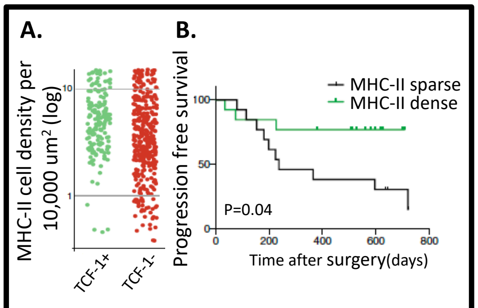

Figure 1. A. Increased density of TCF-1$^+$ T-cells near APCs (MHC-II$^+$ cells). **B.** Increased APC density correlates with improved PFS in renal cell carcinoma.[1]

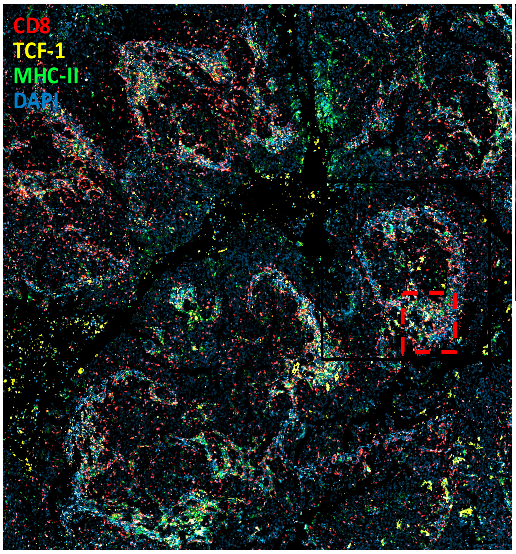

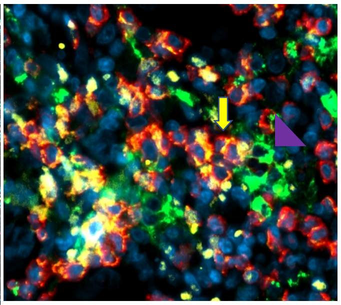

Figure 2. (Left) Large field view of T-cell infiltrates in BM. (Upper Right) Zoom in on immune niche with CD8$^+$ TCF-1$^+$ T-cells (yellow arrow) in close proximity to MHC-II$^+$ cells (purple) forming an immune niche.

Larger brain metastases frequently cause symptoms secondary to cerebral edema. To reduce symptoms prior to resection or SRS treatment, patients are commonly treated with glucocorticoids which are immunosuppressant. Retrospective data suggests concurrent therapeutic glucocorticoid doses reduces an anti-PD-1 stimulated response and patient outcomes.[29,30] Importantly, glucocorticoids have also been shown to negatively impact the development of tertiary lymphoid structures (TLS) in lung cancer.[31] These TLS contain APCs and T-cells similar to our immune niche.[31] We investigated the impact of sustained glucocorticoid administration on the stem-like T-cell population in a murine tumor model using B16F10GP, a melanoma cell line with an inserted lymphocytic choriomeningitis virus (LCMV) protein allowing us to track tumor specific T-cells.[32] We utilized dexamethasone (dex), a potent glucocorticoid, and found a dose dependent reduction in the stem-like intra-tumoral population (**Figure 3**). Our group is now investigating ways to overcome this steroid induced immuno-suppression by using high dose focal radiotherapy similar to SRS.

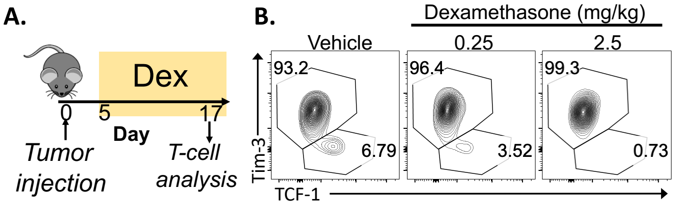

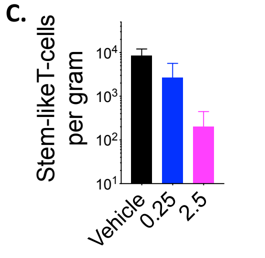

Figure 3. A. Mice injected with 5 x 10^5 B16F10GP murine cell line at Day 0. Dex injected subcutaneously at indicated concentrations on day 5 and continued daily throughout. **B., C.** Gated on tumor-specific T-cells showing decreased frequency and number of stem-like T-cells (Tim-3$^-$ TCF-1$^+$) in tumor at Day 17.

Radiotherapy (RT) is effective as a local treatment, in part, because it enhances T-cell infiltration into irradiated tumors.[33] It is now known to occasionally induce tumor regression outside the radiation field. This impact on tumors outside the RT field occurs via immuno-stimulation, a process known as the abscopal effect.[34-36] RT mediates this, in part, by acting as in-situ vaccine, liberating tumor antigens and generating an inflammatory milieu that enhances the CD8$^+$ T-cell anti-tumor response.[33,37,38] This effect can synergize with checkpoint blockade.[33,39-41] Previously, we demonstrated the ability of radiation to elicit an immune response in our murine tumor model of the abscopal effect.[32] In our studies currently *in press*, single fraction ionizing radiation increases tumor density of stem-like CD8$^+$ T-

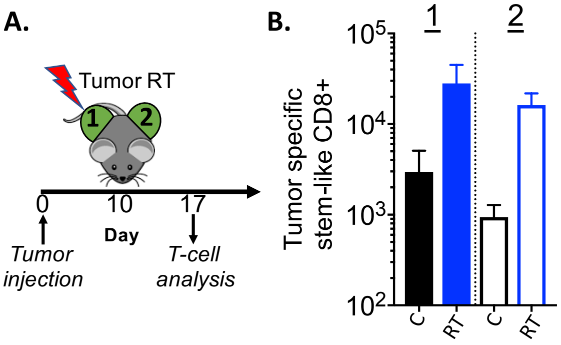

Figure 4. A. schema showing 10 Gy x 1 to primary tumor at Day 10. **B.** increase in stem-like T-cell numbers in (1) irradiated and (2) unirradiated tumor.

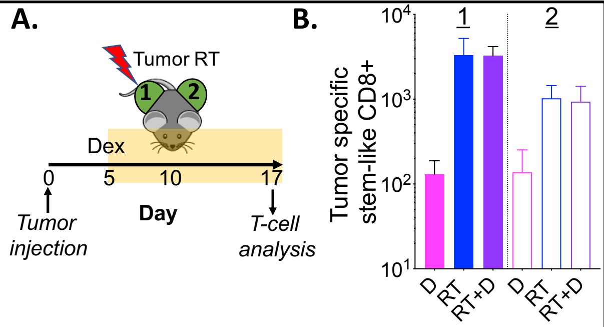

Figure 5. A. Tumors as in Fig 3. Dex (D) (0.5 mg/kg) initiated on Day 5, and RT administered on Day 10 (10 Gy x 1 fraction) to Tumor 1. **B.** Gated on tetramer positive tumor specific population, numbers quantified.

cells. Importantly, single fraction radiation also increases the density of tumor-specific stem-like T-cells in distant, unirradiated tumors, an abscopal effect **(Figure 4)**. Next, we investigated the impact of radiation on dex induced immunosuppression. In preliminary results, we found that the density of stem-like CD8$^+$ T-cells following RT was maintained in the presence of intermediate dose dex (0.5 mg/kg) at the local and distant (abscopal) site (**Figure 5**). ***This suggests that the immunosuppressive effects of dex can be overcome by RT by enhancing intra-tumoral stem-like T-cell numbers and potentially the immune niche density.*** <u>In the following studies, we wish to investigate *whether SRS can enhance niche density in the presence of low or high dose dex*</u>. To this point, we have analyzed resected BM from 10 patients treated with either upfront resection or pre-operative SRS 5-7 days prior to surgery. The pre-operative SRS cohort received >6 mg daily dex for at least 1 week longer that the up-front resection cohort. Despite the larger cumulative dex dose in the pre-op SRS cohort, the density of the immune niche was similar between the two groups (**Figure 6**). This suggests that any reduction in immune niche density caused by dex may be overcome by the immuno-stimulatory activity of SRS. **We hypothesize focused SRS to BM enhances the immune niche density at local and potentially distant intracranial sites and optimizes tumors for an anti-PD-1/L1 response in the presence or absence of dex.**

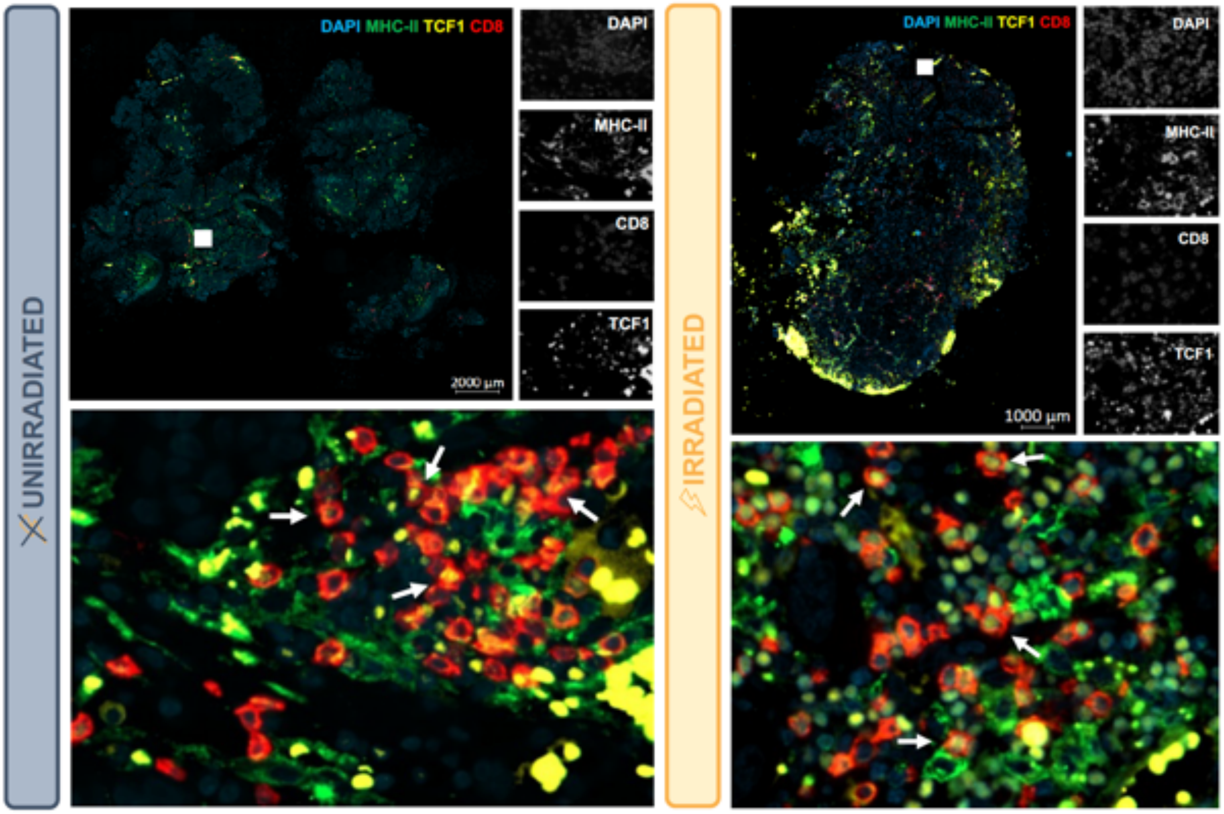

Figure 6. BM (in this case both lung adenocarcinoma) were treated with either upfront resection (left) or pre-operative SRS (15-21 Gy) and 5-7 days later underwent resection. Upper left demonstrates overall immune infiltration; right shows individual stains; bottom demonstrates immune niche with stem-like T-cells and green APC. White boxes highlight area of inset, and white arrows demonstrate TCF-1^{+} stem-like CD8^{+} T-cells.

3.1 Study Rationale

- SRS is important to reduce the risk of local recurrence of brain metastases treated with surgery.
- SRS can be administered pre- or post-operatively, with most institutions administering it post-op.
- Pre-op SRS has the potential to reduce the risk for radiation necrosis and leptomeningeal disease, however, logistical problems exist.
- Surgery + SRS demonstrates a local recurrence rate between 20-25% and distant brain failure is a significant problem.
- Radiation can stimulate an anti-tumor immune response.
- Pre-op SRS will allow for investigation of the impact of radiation and different doses of dexamethasone on the intra-tumoral immune phenotype. This may elucidate ways to utilize radiation to enhance the anti-tumor immune response and improve both local control and distant brain failure.

3.2 Clinical Experience

The purpose of this study is to determine the safety and feasibility of pre-operative SRS at our institution with the secondary objectives described above. The Levine Cancer Center and Dr. Roshan Prabhu, a consultant on this study, have extensive experience in this treatment approach. Dr. Prabhu's group has published extensively on using this treatment regimen. They have previously demonstrated the feasibility of preoperative SRS given as a single fraction prior to planned resection in a cohort of 47 patients.[8] They have also shown that preoperative SRS (in a cohort of 66 patients) has the potential to reduce the risk of radiation necrosis (RN) and leptomeningeal disease (LMD) recurrence with similar cavity LR rates compared with postoperative SRS.[9] Additionally, his group has demonstrated that preoperative SRS is not associated with increased risk of LMD compared with postoperative whole brain RT.[10] His most recently published a retrospective experience demonstrating continued excellent local control and radiation necrosis rates in an updated analysis with increased patient numbers and follow-up time.[11] I contributed to his retrospective study published in 2017 evaluating SRS vs SRS + surgery which included 63 pre-operatively treated patients.[42]

4. Study Intervention/Investigational Agent

This study has three main interventions: 1) SRS to the brain metastasis; 2) Treatment with low vs high dose dexamethasone. This will be administered PO or IV according to treating physician preference with no dose adjustment made for different routes of administration. Dexamethasone will be handled as per standard hospital and outpatient operating procedure. As dexamethasone is part of standard of care for symptomatic BM, insurance will be billed. Intervention is dose adjustment. 3) Resection of the irradiated metastasis.

5. Procedures Involved

Please refer to the study schema (Section 1.2). This is a pilot 2 arm study treating patients with 1-2 brain metastases with pre-operative SRS, dexamethasone and resection. Pre-op SRS dosing is per RTOG 90-05[43] standard dosing with modifications detailed below, and it is based on lesion volume. The neurosurgeon will make the decision about whether the patient is study eligible and a good candidate for resection based on tumor location, associated symptoms, tumor size and need for issue analysis.

During the study, toxicity data will be collected as well as immunologic niche information from the brain metastasis.

5.1 Study Design

This clinical trial is a two-arm pilot study evaluating the safety and feasibility of administering pre-operative SRS at our institution. The study is divided into a Screening period, Treatment period, End of Treatment (EOT) period, and Follow-up period.

During Screening period patients will provide written informed consent to participate in the study before completing any protocol-specified procedures or evaluations not considered to be part of the patient's standard care. Procedures that were performed for standard of care prior to signing informed consent may be used for screening purposes (e.g., full physical exam) as long as the procedures were completed within the **14-day screening period**. After signing the ICF, patients will be evaluated for entry criteria during the screening period (Day -14 to -1). Alternating allocation to study arm A or B starting with Arm A will be administered following screening unless Investigator's decision to unenroll patient or patient withdraws consent. Once enrolled, patients will receive pre-operative SRS on Day 1-14 after consenting

followed by surgical resection of the metastasis on Day 2-21. There will be 3 additional visits for imaging and radiographic assessment of the patient. On study, the patient will be followed for 4 months for toxicity and discharged to standard of care follow-up per treating provider.

5.2 Dosing and Administration

Dose of Dexamethasone in Two Arm Study	
Dose Level	Dose
Arm A (low dose)	≤ 4 mg daily
Arm B (high dose)	≥ 16 mg daily

Agent	Dose	Route	Schedule
Dexamethasone	Range	IV or PO	Range from 2-21 days

5.3 Dose Modification

The investigator and patient will decide whether the patient is able to tolerate the dexamethasone dose on Arm A (low dose) based on neurological symptoms and physical exam. If the patient is unable to tolerate due to severe headaches, worsening focal weakness, seizures or vision changes, the patient will be moved to the high dose arm. Once the high dose arm has completed accrual, patients unable to tolerate the low-dose arm will be ineligible for the study.

5.4 Study Procedures

Before study entry and throughout the study, various clinical and diagnostic laboratory evaluations are outlined. The purpose of obtaining these detailed measurements is to ensure adequate **safety and tolerability assessments**. Clinical evaluations and imaging may be repeated more frequently if clinically indicated. The Schedules of Assessments during the screening and treatment period is provided following the Protocol Synopsis.

Screening Phase

Screening procedures will be performed up to 14 days prior to enrollment and initiation of radiation therapy as applicable, except for baseline imaging (up to 28 days allowed) unless otherwise specified. All subjects must first read, understand, and sign the IRB/REB/IEC-approved ICF before any study-specific screening procedures are performed. After signing the ICF, completing all screening procedures, and being deemed eligible for entry, subjects will be enrolled in the study and allocated sequentially first to the low dose then the high dose dexamethasone arm. Procedures that are performed prior to the signing of the ICF and are considered standard of care may be used as screening assessments if they fall within the screening window.

The following procedures will be performed during the **Screening Visit**:

- Informed Consent
- Review of eligibility criteria
- Medical history and demographics
- Complete physical exam

- ECOG Performance Status
- Vitals signs, weight and height
- Review of prior/concomitant medications
- Imaging by MRI
- Clinical laboratory tests for:
  - Hematology
  - Clinical chemistry
  - Creatinine Clearance
  - Serum or urine pregnancy test (for women of childbearing potential)
  - HIV negative

Treatment Phase

Procedures to be conducted during the treatment phase of the study are presented in the Schedule of Assessments (Section 1.3).

End of Treatment

- End of treatment is defined as immediately following brain metastasis resection.
- Assessments for subjects who have completed treatment are provided in the Schedule of Event.
- All subjects will be followed for survival until the end of the study regardless of further treatments, or until the sponsor ends the study.

5.5 Description of Study Procedures

Medical history

Findings from medical history (obtained at screening) and physical examination shall be given a baseline grade according to the procedure for AEs. Increases in severity of pre-existing conditions during the study will be considered AEs, with resolution occurring when the grade returns to the pre-study grade or below.

Physical examination

Physical examinations should be conducted according to the Schedule of Events. Full physical examinations should be conducted at screening/baseline, at first follow-up and beyond, and EOT (evaluate all major organ systems, including the following categories: general, head, eyes, ears, mouth/throat, neck, heart, lungs, abdomen, lymph nodes, joints, extremities, integumentary, neurologic, and psychiatric). Other examinations may be focused, at the discretion of the Investigator, to identify changes from baseline or evaluate changes based on the patient’s clinical symptoms. Weight is to be reported at each visit, height at screening/baseline visit only.

Vital signs

Vital signs (blood pressure [BP], pulse, temperature, and respiration rate) will be evaluated according to the assessment schedules. Body weight is also recorded along with vital signs.

Dexamethasone

Dexamethasone will be administered PO or IV at either ≤ 4 mg daily or ≥ 16 mg daily from enrollment until resection according to enrollment arm.

Delivery of Radiation Treatment

Radiation Simulation and Diagnostic Procedures

All simulation and treatment procedures represent current institutional practice and will be the same for all study participants.

CT Simulation: A CT simulation will be performed for radiation therapy treatment planning purposes several days to the initiation of radiation therapy. This procedure consists of a CT scan performed in the treatment position. It is not for diagnostic purposes and is not itself therapeutic, but the CT image is required for radiation planning and delivery. This procedure is standard of care prior to therapeutic radiation. The CT simulation will occur with the patient supine position in a framed and/or frameless thermoplastic head mask at discretion of treating physician.
-CT simulation scan slice thickness may not exceed 1.25 mm

Pre-treatment MRI of the brain: A high resolution MRI with and without gadolinium contrast for treatment planning will be acquired within 28 days before treatment delivery. This planning MRI is standard of care for patients with intracranial metastases planned for SRS.
-Treatment planning MRI slice thickness may not exceed 3 mm.

LINAC, SRS Based Radiation Therapy, Required Criteria

1. Treatment shall be delivered with megavoltage machines of a minimum energy of 4 MV photons. Selection of the appropriate photon energy should be based on optimizing the radiation dose distribution within the target volume and minimizing dose to non-target normal tissue.
2. LINAC treatment should also include isocentric conical collimators, mini-multi-leaf (5 mm or less) technology or linear accelerators mounted on robotic arms.
3. Either a framed or frameless stereotactic, relocatable immobilization system will be used for treatment simulation and delivery. These systems may include modified stereotactic frames, camera-based localization systems, etc. The immobilization/ relocalization system should be capable of reproducing the patient setup to within 3 mm.
4. Single fraction radiosurgery treatment may be delivered with intensity modulated radiation surgery (IMRS) or dynamic conformal arcs (DCA). Multiple fraction radiation treatment can be delivered by intensity modulated radiation therapy (IMRT) or volume modulated arc therapy (VMAT).
5. Patients must be positioned for each treatment using 3-dimensional imaging i.e. **Cone Beam CT**. Cone beam CT scans with each radiation fraction on the treatment machine will be performed daily prior to SRS with the patient in the treatment position to assure accurate repositioning of the PTVs.
6. Radiation therapy will consist of 1-3 fractions. All radiation treatment fractions should be completed by end of week 2 of treatment.
7. Multiple vertex and coplanar/noncoplanar beams should be used and arranged with the goal of excluding as much normal brain tissue as possible outside of the PTV at high and intermediate dose levels.

Target Volume Determination

1. The gross tumor volume (GTV) will be defined by as the MRI defined T1 post contrast enhancing brain metastasis. Surrounding areas of edema will not be considered part of the target volume
2. The planning target volume (PTV) will be the GTV + 1 mm margins.

Dose prescription and dosimetry requirements

1. Dose will be prescribed to the isodose line that encompasses the entirety of the PTV. Treatment planning and dose acceptance will be per institutional standard of care.

   A. If intensity modulated radiosurgery (IMRS) is utilized, standard prescription isodose is to 98% (range 95-100%, acceptable)

   B. If dynamic conformal arcs (DCA) are utilized, standard prescription isodose is to 80% (range 70-90%, acceptable).

2. Doses are specified such that at least 95% of the PTV shall receive 100% of the prescribed dose.
3. The marginal dose and the 100% dose (isocenter dose) may be recorded for each patient.
4. For quality control, representative isodose lines (e.g. 20%, 40%, 60%, 80%, 90%) may be generated for each patient.

Quality of PTV coverage will be categorized according to selected isodose line:

   A. Total – selected isodose line completely encompasses the PTV
   B. Marginal – selected isodose line incompletely encompasses the PTV, but not by more than 10% of the PTV volume.
   C. Subtotal – selected isodose line incompletely encompasses the PTV, but by more than 10% of the PTV volume. This will not be accepted.

5. Conformality index requirements (ratio of prescription isodose volume to the target volume *(PI/TV)*)

   A. Per protocol if between 1.0 and 2.0, acceptable
   B. Acceptable variation if ≥ 0.9 but < 1.0 or >2.0 but ≤ 3.5.
   C. Unacceptable deviation if > 3.5.

Dose Limitation to Critical Structures

1. In addition to the above defined GTVs and PTVs, both eyes, the lenses of both eyes, the optic nerves, the optic chiasm, cochlea, the brainstem, and the spinal cord must be evaluated per standard of care approaches. Dose-volume histograms will be generated and whole organ dose and maximum point dose will be recorded for each critical structure. Dose limitations to normal structures are defined below.
2. If the patient has received no prior cranial irradiation, the maximal point doses permissible to the structures from the current radiation therapy plan are listed below.
3. Patient cannot have received WBRT or SRS prior to being enrolled on this trial. However, once on the trial, patient can have received multiple SRS and or whole brain radiation as part of the standard of care treatments for progressive disease.

Radiation Dose

Preferred Regimen

Max Diameter	Tumor Volume (cm^3)	Number of Fractions	Dose per fraction (Gy)
0 – 2 cm	<4.2	1	21
2 – 2.5 cm	4.2-8.2	1	18
2.5 – 3 cm	8.2 – 14.2	1 or 3	18 or 27 based on V12 normal brain V12 ≥ 10 cc
3 – 4 cm	14.2 – 33.5	3	27
> 4 cm	> 33.5	3	24

Alternate dosing if neurosurgery is not able to accommodate a fractionated course

Max Diameter	Tumor Volume (cm^3)	Number of Fractions	Dose per fraction (Gy)
2.5 – 3	8.2 – 14.2	1	15 – 16 based on V12 normal brain V12 ≥ 10 cc
3 – 3.5 cm	14.2 – 22.5	1	14
3.5 – 4 cm	22.5 – 33.5	1	13
> 4 cm	> 33.5	1	12-13

Normal Tissue Constraints by number of fractions

Normal Tissue Constraints, 3 Radiation Fraction		
**Structure**	**Volume**	**Volume**
Brainstem	Max ≤ 0.03cc	18
Spinal Cord	Max ≤ 0.03cc	18
Optic Nerve, each	Max ≤ 0.03cc	15
Optic Chiasm	Max ≤ 0.03cc	15
Cochlea	Max ≤ 0.03cc	20
Lens	Max ≤ 0.03cc	4
Eye (Globe), each	Max ≤ 0.03cc	18
**Normal Tissue Constraints, 1 Radiation Fraction**		
**Structure**	**Volume**	**Volume**
Brainstem	Max ≤ 0.03cc	10
Spinal Cord	Max ≤ 0.03cc	18
Optic Nerve, each	Max ≤ 0.03cc	8
Optic Chiasm	Max ≤ 0.03cc	8
Cochlea	Max ≤ 0.03cc	12
Lens	Max ≤ 0.03cc	2.5
Eye (Globe), each	Max ≤ 0.03cc	16
Normal Brain	10cc	12 Gy

Surgical Resection

Operative intervention and brain metastasis(es) resection will be done per standard operative procedures.

6. Data and Specimen Banking

Tumor samples will be obtained and used for medical research by the investigators of this study. Data and specimens from this study may be useful for other research being done by investigators at Emory or elsewhere. To help further science, Investigators may provide de-identified data and/or specimens to other researchers. Any information that could identify participants will not be included. If data or specimens are labeled with study ID, we will not allow other investigators to link that ID to identifiable information. The tumor specimens will be analyzed by flow cytometry with markers including, but not be limited to CD8, TCF-1, CD44, Ki-67, CD4, PD-1, and immunofluorescence as described above.

Blood samples will be obtained at *Visit 0, 3, 5, and 6. Samples will be drawn into three* dark green top tubes with heparin *for PBMC and plasma isolation. Tubes will be labeled and logged at Emory's Winship Cancer Institute by a skilled clinical research nurse or phlebotomist. They will be maintained at room temperature until transport to Dr.* Buchwald'*s laboratory. Samples distributed to the* Buchwald *laboratory will only be identifiable by an assigned donor number, study identifier and a draw date. Samples will be transported in sealed biohazard containers between sites per standard protocol. Whole blood and/or PBMC will be used fresh or will be frozen and banked for future batch analyses. Plasma will be frozen at -80C. Frozen PBMC samples will be stored in liquid nitrogen* in the Buchwald laboratory*. All assays will be performed as per* Buchwald *lab standard of practice. Briefly, frequency and absolute cell counts will be determined for the major lymphocyte populations (CD3, CD4, CD8, CD19) and monocytes (CD14) through the use of BD TruCount tubes. Detailed phenotypic analysis will be performed through whole blood staining with the following markers; CD3, CD4, CD8, Foxp3, CD45RA, CCR7, CD28, CD27, CD127, PD-1, Ki-67, Bcl-2, HLA-DR, CD38, ICOS, CD137, Tbet, eomes, Granzyme B, Perforin, CTLA-4, Tim-3, CD14, CD16, CD11c, CD123, PD-L1, CD86. HLA-A2 positive patients will be monitored for the presence and activation of MART-1 and NY-ESO-1-specific CD8 T cells. Additional phenotypic, genomic and/or proteomic analysis may be performed on banked PBMC samples. Besides flow cytometry phenotypic analysis, sample collected at early time points after irradiation, will be used for transcriptional profiling. Frozen plasma will be used for monitoring cytokine expression and/or possibly other translational* analysis.

Samples and toxicity data collected under this protocol will be stored. The purified PBMC will be stored at -80 degrees in the Buchwald laboratory. The fixed tissue will also be stored in the Buchwald lab at 4 degrees. Access to stored samples will be limited to IRB-approved investigators and requires an ID badge and a key to the locked -80 or 4 degree. Data will be stored using codes assigned by the investigators or their designees. Data will be kept in password-protected computers. Only investigators will have access to the samples and data.

All stored samples will be maintained in the laboratory to which it was sent initially for analysis. Study participants who request destruction of samples will be notified of compliance with such request and all supporting details will be maintained for tracking.

The results of some study tests and procedures will be used only for research purposes and will not be placed in subject's medical record. For this study, those items include: brain metastasis tissue.

7. Sharing of Results with Participants

In general, study staff will not provide any individual results to subjects (ex. outcome trial results or results from subject's samples studies). If something of urgent medical importance to the participating subjects will be found, the PI (or co-Is) will inform the subject, although we expect that this will be a very rare occurrence. Samples and data will only be used for research.

8. Study Timelines

From enrollment through treatment and follow-up the patient will be on-study for 4 months. The patient will continue to be followed per the standard of care, but outside the study context.

8.1 Duration of therapy

In the absence of treatment delays due to adverse event(s), patients will undergo treatment unless the following:

• Death
• Symptomatic deterioration
• Investigator's decision to not treat
• Patient decision to discontinue treatment
• Patient withdraws consent
• Lost to follow up
In the event of a patient's withdrawal, the Investigator will make every effort to complete the End of Treatment procedures specified in the Schedule of Events.

8.2 Duration of follow-up

Patients will be followed for approximately 120 days (Safety Follow-up) after the single fraction of SRS or before initiation of new antineoplastic or investigational therapy whichever occurs first.

Long-term follow-up should continue until the patient's withdrawal of consent or loss to follow up, death, or study termination.

Patient records may be reviewed until death to assess progression and survival. Survival information may be collected by clinic visit, email, or telephone after ending protocol treatment and until the study is terminated, the patient dies, or the patient is lost to follow-up.

A participant will be considered lost to follow-up if he fails to return for three scheduled visits and is unable to be contacted by the study site staff after three attempts at contact by phone. The following actions must be taken if a participant fails to return to the clinic for a required study visit: The site will attempt to contact the participant and reschedule the missed visit and counsel the participant on the importance of maintaining the assigned visit schedule and ascertain if the participant wishes to and/or should continue in the study.

Patients who have not initiated a new antineoplastic regimen will have the following assessments:
• Radiologic tumor assessments every 12 weeks (± 7 days)
• In case of a clinically significant AE, patient will be followed for safety until resolution or permanent sequelae of all toxicities attributable to treatment.

9. Inclusion and Exclusion Criteria

Inclusion Criteria

1. Age ≥18 years.
2. Prior pathologically confirmed or suspected extracranial diagnosis of malignancy
3. Brain metastases visible on contrasted MRI brain
4. ECOG performance status ≤2 (Karnofsky ≥60%, see Appendix A).
5. Life expectancy > 12 weeks as determined by the Investigator
6. Patients must have adequate organ function as determined by Neurosurgery to undergo surgery
7. Willingness and ability of the subject to comply with scheduled visits, study procedures, and study restrictions.
8. Evidence of a personally signed informed consent indicating that the subject is aware of the neoplastic nature of the disease and has been informed of the procedures to be followed, the experimental nature of the therapy, alternatives, potential risks and discomforts, potential benefits, and other pertinent aspects of study participation.
9. Patient must have a negative pregnancy test, be actively taking oral contraceptives or have undergone a hysterectomy.

Exclusion criteria

An individual who meets any of the following criteria will be excluded from participation in this study:

1. Patients on any immunosuppressive medication other than dexamethasone
2. Patients who are receiving any other investigational agents or an investigational device within 21 days before administration of first dose of study drugs.
3. Uncontrolled intercurrent illness including, but not limited to, ongoing or active infection, symptomatic congestive heart failure, unstable angina pectoris, cardiac arrhythmia, or psychiatric illness/social situations that would limit compliance with study requirements.
4. HIV-positive
5. Pregnant or nursing women are excluded
6. Prior whole brain radiotherapy or SRS to the same site planned for surgery

10. Local Number of Participants

We will be recruiting 20 participants at Winship (10 on Arm A, 10 on Arm B). Patients will be registered after signing of the informed consent document and meeting all entry requirements.

11. Recruitment Methods

Investigators, nurses, and/or data managers review lists of cancer patients who have cancer and will determine if there are patients who might be eligible for a clinical trial. The nurse/data manager reviews accessible medical records to screen further for eligibility. The nurse reviews the eligibility with the physician.
Subjects will be identified by their treating physicians. Clinical care team at Winship will inform potential subjects about the known benefits and potential risks of a clinical trial as well as other available treatment options.
Some of the subjects recruited for this protocol will be patients being treated at Emory and under the care of one or more of the study investigators. Some potential subjects will be identified by their treating physician and referred to Emory for possible participation in the protocol.

No incentives are provided to patients for trial participation.

Study personnel will notify Winship Central Subject Registration (WCSR) by email at winshipcsr@emory.edu, once subject has been consented for a trial.
Email notification must be done within 24 hours after consent has been obtained and it will include scanned copies of:

- Signed patient consent form
- HIPAA authorization form
- Emory Research Management System (ERMS; *https://erms.emory.edu*) Enrollment Fax Cover

The WCSR will enter the subject into the OnCore Research Management System, which is the system of record for Winship Cancer Institute Clinical Trials.

Enrolling a subject requires careful screening and determination of eligibility.

Eligible patients will be enrolled on study centrally at Winship Cancer Institute by the Study Coordinator. When all required imaging results are available, complete the eligibility checklist and provide the checklist and the supporting documentation to the IRB approved investigator for review and sign-off. Once the investigator (sub-investigator, Co-Investigator) has signed the eligibility checklist enrollment may proceed. Oncore and ERMS must be updated to reflect eligibility and on treatment status.

Following enrollment, patients should begin protocol treatment within_____5_____ business days. Issues that would cause treatment delays should be discussed with the Principal Investigator.

12. Withdrawal of Participants

Participants are free to withdraw from participation in the study at any time upon request.
An investigator may discontinue or withdraw a participant from the study for the following reasons:

- Pregnancy
- Significant study intervention non-compliance
- If any clinical adverse event (AE), laboratory abnormality, or other medical condition or situation occurs such that continued participation in the study would not be in the best interest of the participant
- Disease progression which prevents administration of the study intervention
- If the participant meets an exclusion criterion (either newly developed or not previously recognized) that precludes further study participation

The reason for participant discontinuation or withdrawal from the study will be recorded on the Case Report Form (CRF).

13. Risks to Participants

- **Neurological symptoms** – Patients will be allocated to either the low or high dose dexamethasone arm. The low dose arm may increase the patient's neurological symptoms including headaches, vision symptoms, seizures and/or focal weakness. If symptoms are deemed significant according to patient or investigator, the patient will be allocated to the high dose arm.

- **Data security-** Subjects will be asked to provide personal health information (PHI). All attempts will be made to keep this PHI confidential within the limits of the law. However, there is a chance that unauthorized persons will see the subjects' PHI. All records will be kept in a locked file cabinet or

maintained in a locked room at the participating sites. Electronic files will be password protected behind an academic institutional firewall. Only people who are involved in the conduct, oversight, monitoring, or auditing of this study will be allowed access to the PHI that is collected. Any publications from this study will not use information that will identify subjects. Organizations that may inspect and/or copy research records maintained at the participating sites for quality assurance and data analysis include groups such as the National Cancer Institute (NCI) and Food and Drug Administration (FDA).

14. Potential Benefits to Participants

Pre-operative SRS has demonstrated lower rates of leptomeningeal disease in retrospective analyses, a significant cause of morbidity and mortality.

15. Data Management and Confidentiality

15.1 Statistical Analysis Plan

This is a non-randomized, two-arm, single-institution pilot study of pre-operative SRS to assess safety. 20 patients will be enrolled in alternating sequence starting on Arm A and then Arm B. The total enrollment will be 10 per arm.

15.1.1 Primary Endpoint

Safety: The primary objective of this study is to describe the safety in terms of grade > 3 CNS toxicity at 4 months. Tolerability of this regimen will be defined as <33% of patients develop grade > 3 at 4 months. This interval is based on (1) unusual radiation related adverse effects beginning as early as 4 months post SRS, and (2) typical timeline used in other trials.

The primary endpoint will be analyzed separately for arms A and B. Adverse events will be summarized descriptively using frequencies and percentages.

15.1.2 Secondary Endpoints

Niche Density: Niche density will be assessed by immunofluorescence as described above. It will be summarized descriptively as the percent of tumor occupied by immune niche for each treatment arm, and will be compared between Arms A and B using a two-sample t-test or non-parametric equivalent, such as Mann-Whitney U test.

Time to Local Recurrence (LR): Time to LR is defined as time from pre-operative SRS to intracranial progression at the treated site. Those without intracranial progression at the treated site will be censored at last follow-up date or death. LR will be estimated using the Kaplan-Meier method, and a 95% confidence interval for median LR will be estimated using the Brookmeyer-Crowley approach.

Time to Anywhere Brain Failure (ABF): Time to ABF is defined as time from pre-operative SRS to intracranial progression at any site within the brain. Those without intracranial progression at any site will be censored at last follow-up date or death. ABF will be estimated using the Kaplan-Meier method, and a 95% confidence interval for median TBF will be estimated using the Brookmeyer-Crowley approach.

Overall survival (OS): Overall survival is defined as time from pre-operative SRS initiation to death. Those alive will be censored at date of last follow-up. OS will be

estimated using the Kaplan-Meier method, and a 95% confidence interval for median OS will be estimated using the Brookmeyer-Crowley approach.

15.1.3 Sample Size and Power Calculations

This is a non-randomized, two-arm pilot study to assess pre-operative SRS. The safety profile should be well-characterized with 10 patients in each cohort. If the true toxicity event rate is 10%, then there is a 7.0% chance of observing 3 or more toxicity events out of 10 patients in either arm.

15.1.4 Analysis populations

Efficacy population: All subjects enrolled in the study who receive pre-operative SRS.
Safety population: All subjects enrolled in the study who receive pre-operative SRS.

15.1.5 Interim analysis

Following the enrollment of the first 6 patients (3/arm), if 2 (33%) experience Grade $\geq$ 3 CNS toxicity listed the 4-month time point, enrollment will be stopped and evaluated by the Data and Safety Monitoring Committee.

15.2 Data/specimens:

Participant confidentiality is strictly held in trust by the participating investigators, their staff, and the sponsor(s) and their agents. Therefore, the study protocol, documentation, data, and all other information generated will be held in strict confidence. No information concerning the study or the data will be released to any unauthorized third party without prior written approval of the Principal Investigator. The study participant's contact information will be securely stored at each clinical site for internal use during the study. At the end of the study, all records will continue to be kept in a secure location for as long a period as dictated by local IRB and Institutional regulations.
Data and/or data forms will be submitted in the clinical management system - Online Collaborative Research Environment (ONCORE)- per Winship SOP 4.2 Data Completion Metrics.
All information in original records and certified copies of original records or clinical findings, observations, or other activities necessary for the reconstruction and evaluation of the trial is considered source data. Source data are contained in source documents, which can be original records or certified copies of hospital records, clinical and office charts, laboratory notes, memoranda, subjects' diaries of evaluation checklists, pharmacy dispensing records, recorded data from automated instruments, copies or transcriptions certified after verification as being accurate and complete, microfiches, photographic negatives, microfilm or magnetic media, x-rays, subject files, and records kept at the pharmacy, at the laboratories, and at medico-technical departments involved in the clinical trial. Case Report Forms (CRFs) – could be used as a tool for collecting and recording data in source documents.

All documentation of adverse events, records of study drug receipt and dispensation, and all IRB correspondence will be maintained for at least 2 years after the investigation is completed.

Study participant research data, which is for purposes of statistical analysis and scientific reporting, will be stored. This will not include the participant's contact or identifying information. Rather, individual participants and their research data will be identified by a unique study identification number. The study data entry and study management systems used by clinical sites and research staff will be secured and password protected. At the end of the study, all study databases will be de-identified and archived.

Samples and data collected under this protocol may be used to study brain metastases. Access to stored samples will be limited to IRB-approved investigators. Samples and data will be stored using codes

assigned by the investigators or their designees. Data will be kept in password-protected computers. Only investigators will have access to the samples and data.

All stored samples will be maintained in the laboratory to which it was sent initially for analysis. Study participants who request destruction of samples will be notified of compliance with such request and all supporting details will be maintained for tracking

16. Provisions to Monitor the Data to Ensure the Safety of Participants

This protocol represents a change in the sequencing of standard of care therapies. Currently, a number of tertiary care centers already employ this sequencing without any increase in toxicity. We, therefore, do not anticipate any additional toxicity over the standard of care. Nevertheless, below are detailed possible AEs and SAEs.

Definition of Adverse Events (AE)
Adverse event means any untoward medical occurrence associated with the use of an intervention in humans, whether or not considered intervention-related (21 CFR 312.32 (a)).

Definition of Serious Adverse Events (SAE)

An adverse event (AE) or suspected adverse reaction is considered "serious" if, in the view of either the investigator or sponsor, it results in any of the following outcomes:

- Death
- Life-threatening adverse event
- Inpatient hospitalization or prolongation of existing hospitalization
- A persistent or significant incapacity or substantial disruption of the ability to conduct normal life functions, or a congenital anomaly/birth defect.
- Important medical events that may not result in death, be life-threatening, or require hospitalization may be considered serious when, based upon appropriate medical judgment, they may jeopardize the participant and may require medical or surgical intervention to prevent one of the outcomes listed in this definition. (Examples of such medical events include allergic bronchospasm requiring intensive treatment in an emergency room or at home, blood dyscrasias or convulsions that do not result in inpatient hospitalization, or the development of drug dependency or drug abuse).

Classification of an Adverse Event

<u>Severity of Event</u>

For adverse events (AEs) not included in the protocol defined grading system, the following guidelines will be used to describe severity.

- **Mild** – Events require minimal or no treatment and do not interfere with the participant's daily activities.
- **Moderate** – Events result in a low level of inconvenience or concern with the therapeutic measures. Moderate events may cause some interference with functioning.
- **Severe** – Events interrupt a participant's usual daily activity and may require systemic drug therapy or other treatment. Severe events are usually potentially life-threatening or incapacitating. Of note, the term "severe" does not necessarily equate to "serious".

Relationship to Study Intervention

All adverse events (AEs) must have their relationship to study intervention assessed by the clinician who examines and evaluates the participant based on temporal relationship and his/her clinical judgment. The degree of certainty about causality will be graded using the categories below. In a clinical trial, the study product must always be suspect.

- **Definitely Related** – There is clear evidence to suggest a causal relationship, and other possible contributing factors can be ruled out. The clinical event, including an abnormal laboratory test result, occurs in a plausible time relationship to study intervention administration and cannot be explained by concurrent disease or other drugs or chemicals. The response to withdrawal of the study intervention (dechallenge) should be clinically plausible. The event must be pharmacologically or phenomenologically definitive, with use of a satisfactory rechallenge procedure if necessary.
- **Probably Related** – There is evidence to suggest a causal relationship, and the influence of other factors is unlikely. The clinical event, including an abnormal laboratory test result, occurs within a reasonable time after administration of the study intervention, is unlikely to be attributed to concurrent disease or other drugs or chemicals, and follows a clinically reasonable response on withdrawal (dechallenge). Rechallenge information is not required to fulfill this definition.
- **Potentially Related** – There is some evidence to suggest a causal relationship (e.g., the event occurred within a reasonable time after administration of the trial medication). However, other factors may have contributed to the event (e.g., the participant's clinical condition, other concomitant events). Although an AE may rate only as "possibly related" soon after discovery, it can be flagged as requiring more information and later be upgraded to "probably related" or "definitely related", as appropriate.
- **Unlikely to be related** – A clinical event, including an abnormal laboratory test result, whose temporal relationship to study intervention administration makes a causal relationship improbable (e.g., the event did not occur within a reasonable time after administration of the study intervention) and in which other drugs or chemicals or underlying disease provides plausible explanations (e.g., the participant's clinical condition, other concomitant treatments).
- **Not Related** – The AE is completely independent of study intervention administration, and/or evidence exists that the event is definitely related to another etiology. There must be an alternative, definitive etiology documented by the clinician.

Adverse Event and Serious Adverse Event Reporting

Expectedness

Principal Investigator will be responsible for determining whether an adverse event (AE) is expected or unexpected. An AE will be considered unexpected if the nature, severity, or frequency of the event is not consistent with the risk information previously described for the study intervention.

Adverse Event Reporting

From the time of treatment allocation/randomization through **120** days following cessation of treatment, all adverse events, that begin or worsen after informed consent, **must be recorded** by the investigator or designee at each examination on the Adverse Event case report forms/worksheets.

The investigator will make every attempt to follow all subjects with non-serious adverse events for outcome.

Conditions that were already present at the time of informed consent should be recorded in the Medical History page of the patient's CRF/worksheet.

Adverse events will be assessed and graded according to the Common Terminology Criteria for Adverse Events (CTCAE) version 5.0. Grade 1 to 5 will be used to characterize the severity of the Adverse Event. If CTCAE grading does not exist for an adverse event, the severity of mild, moderate, severe, and life-threatening, death related to the AE corresponding respectively to Grades 1 - 5, will be used. Information about any deaths (related to an Adverse Event or not) will also be collected through a Death form (or EOT/SEC/Survival Information in NOVDD). The occurrence of adverse events should be sought by non-directive questioning of the patient (patient) during the screening process after signing informed consent and at each visit during the study. Adverse events also may be detected when they are volunteered by the patient (patient) during the screening process or between visits, or through physical examination, laboratory test, or other assessments. As far as possible, each adverse event should be evaluated to determine:

1. The severity grade (CTCAE Grade 1-5)
2. Its duration (Start and end dates)
3. Its relationship to the study treatment (Reasonable possibility that AE is related: No, Yes) or Its relationship to the study treatment (Reasonable possibility that AE is related: No, Yes, investigational treatment, Yes, the study treatment (non-investigational), Yes, both and/or indistinguishable)
4. Action taken with respect to study or investigational treatment (none, dose adjusted, temporarily interrupted, permanently discontinued, unknown, not applicable)
5. Whether medication or therapy was given (no concomitant medication/non-drug therapy, concomitant medication/non-drug therapy)
6. Whether it is serious, where a serious adverse event (SAE) is defined as in Section 9.2 and which seriousness criteria have been met (include for NCDS trials).
7. Outcome (not recovered/not resolved, recovered/resolved, recovering/resolving, recovered/resolved with sequelae, fatal, unknown)

If the event worsens the event should be reported a second time in the CRF noting the start date when the event worsens in toxicity. For grade 3 and 4 adverse events only, if improvement to a lower grade is determined a new entry for this event should be reported in the CRF noting the start date when the event improved from having been Grade 3 or Grade 4. All adverse events should be treated appropriately (see below for specific management). If a concomitant medication or non-drug therapy is given, this action should be recorded on the Adverse Event CRF.
Once an adverse event is detected, it should be followed until its resolution or until it is judged to be permanent, and assessment should be made at each visit (or more frequently, if necessary) of any changes in severity, the suspected relationship to the study treatment, the interventions required to treat it, and the outcome. Progression of malignancy (including fatal outcomes), if documented by use of appropriate method (for example, as per RECIST criteria for solid tumors), should not be reported as a serious adverse event.
Adverse events separate from the progression of malignancy (example, deep vein thrombosis at the time of progression or hemoptysis concurrent with finding of disease progression) will be reported as per usual guidelines used for such events with proper attribution regarding relatedness to the drug.
Laboratory abnormalities that constitute an Adverse event in their own right (are considered clinically significant, induce clinical signs or symptoms, require concomitant therapy or require changes in study treatment), should be recorded on the Adverse Events CRF.
Laboratory abnormalities, that do not meet the definition of an adverse event, should not be reported as adverse events. A Grade 3 or 4 event (severe) as per CTCAE does not automatically indicate a SAE unless it meets the definition of serious as defined below and/or as per investigator's discretion. A dose

hold or medication for the lab abnormality may be required by the protocol in which case the lab abnormality would still, by definition, be an adverse event and must be reported as such.

Dose limiting toxicities

Dose limiting toxicity definition

Dose limiting toxicity is defined as a Grade 3 or higher CTCAE CNS toxicity at 4 months from first SRS treatment. Below is a table of the most common CNS toxicities.

Adverse Event	Grade 1	Grade 2	Grade 3	Grade 4	Grade 5
**Central Nervous System Necrosis**	Asymptomatic; clinical or diagnostic observations only; intervention not indicated	Moderate symptoms; corticosteroids indicated	Severe symptoms; medical intervention indicated	Life-threatening consequences; urgent intervention indicated	Death
**Seizure** (Definition: A disorder characterized by a sudden, involuntary skeletal muscular contractions of cerebral or brain stem origin.)	Brief partial seizure; no loss of consciousness	Brief generalized seizure	Multiple seizures despite medical intervention	Life-threatening; prolonged repetitive seizures repetitive seizures	Death
**Cognitive Impairment**	Mild inattention or decreased level of concentration	Moderate impairment in attention or decreased level of concentration; limiting instrumental ADL	Severe impairment in attention or decreased level of concentration; limiting self care ADL		
**Cognitive Disturbance**	Mild cognitive disability; not interfering with work/school/life performance; specialized educational services/devices not indicated	Moderate cognitive disability; interfering with work/school/life performance but capable of independent living; specialized resources on part time basis indicated	Severe cognitive disability; significant impairment of work/school/life performance		
**Stroke**	Asymptomatic or mild neurologic deficit; radiographic findings only	Moderate neurologic deficit	Severe neurologic deficit	Life-threatening consequences; urgent	Death

				intervention indicated	
**Intracranial Hemorrhage** (A disorder characterized by bleeding from the cranium.)	Asymptomatic; clinical or diagnostic observations only; intervention not indicated	Moderate symptoms; medical intervention indicated	Ventriculostomy, ICP, monitoring, intraventricular thrombolysis, or operative intervention indicated	Life-threatening consequence s; urgent intervention indicated	Death
**Headache** (definition: A disorder characterized by a sensation of marked discomfort in various parts of the head, not confined to the area of distribution of any nerve.)	Mild Pain	Moderate pain, limiting instrumental ADL	Severe pain, limiting self-care ADL		
**Wound Infection**	Localized, local intervention indicated	Oral intervention indicated (e.g. antibiotics, antifungal or antiviral)	IV antibiotics, antifungal or antiviral; invasive intervention indicated	Life-threatening consequence ; urgen intervention indicated	Death

<u>CNS Toxicities and Intervention</u>

Central Nervous System Necrosis:

o For **Grade 2 events**, treat with systemic steroids, pentoxyfilline and vitamin E. Consider starting with doses less than dexamethasone 6mg PO daily (2 mg TID), if clinically acceptable and indicated.
o For **Grade 3-4 events**, immediately treat with intravenous steroids. Administer additional anti-inflammatory measures, such as hyperbaric oxygen and/or surgical intervention.
o When symptoms improve to Grade 1 or less, steroid taper should be started immediately, continued over no less than 4 weeks.
o It may be that a patient may need low doses of steroids long term, in that case, the goal is to achieve the lowest tolerated steroid levels to manage the radiation necrosis.

Seizure:

o For **Grade 1-2** events, treat with oral anti-seizure medications. If clinically safe and acceptable, consider starting with levetiracetam 1000mg BID, or alternative.

o For **Grade 3-4** events, treat with oral and/or IV anti corticosteroids with consideration for surgery, if clinically feasible.
o When symptoms improve to Grade 1 or less, steroid taper should be started and continued over no less than 4 weeks.

Cognitive Impairment or Cognitive Disturbance

o For **Grade 2 events**, treat with systemic steroids. Consider starting with doses less than 6 mg PO dexamethasone (2 mg TID), if clinically acceptable and indicated. Memantine or other Alzheimer related medication can also be considered.
o For **Grade 3-4 events**, immediately treat with intravenous steroids.
o When symptoms improve to Grade 1 or less, steroid taper should be started and continued over no less than 4 weeks.

Clinical Criteria for Early Trial Termination

Although a number of tertiary centers perform pre-operative SRS followed by surgical resection, our current institutional standard is post-operative SRS. Therefore, early termination will be dictated, in part, by the Adverse Event rates seen with post-op SRS.[12] Following the enrollment of the first 6 patients including both arms, if 2 (33%) experience Grade $\geq$ 3 CNS toxicity listed above at the 4 month time point, enrollment will be stopped and evaluated by the Data and Safety Monitoring Committee.

Addition criteria for early termination:

1. Quality and quantity of data recording is inaccurate or incomplete
2. Poor adherence to protocol and regulatory requirements
3. Incidence or severity of toxicity from pre-operative radiation is significantly greater that resection followed by post-operative SRS as detailed above.

Intracranial Disease Progression on Study

Should the enrollee develop intracranial disease progression during the study's 4-month follow-up period they are eligible to remain on study and receive standard of care treatment which may include additional SRS or whole brain radiotherapy.

Serious Adverse Event Reporting

For the time period beginning at treatment allocation/randomization through **120** days following cessation of treatment, any serious adverse event, or follow up to a serious adverse event, including death due to any cause whether or not related to the study treatment, must be **submitted on an SAE form** and assessed by PI in order to determine reporting criteria to regulatory authorities, IRB, DSMC, FDA or Sponsor as applicable.
All SAEs will be followed until satisfactory resolution or until the site investigator deems the event to be chronic or the adherence to be stable. Other supporting documentation of the event may be requested by the study sponsor and should be provided as soon as possible.
All subjects with serious adverse events must be followed up for outcome. Any additional information for the SAE including complications, progression of the initial SAE, and recurrent episodes must be reported as follow-up to the original episode **within 24 hours** of the investigator receiving the follow-up information.
An SAE occurring at a different time interval or otherwise considered completely unrelated to a previously reported one should be reported separately as a new event.
Any SAEs experienced after the reporting period described above should only be reported to FDA/IRB

if the investigator suspects a causal relationship to the study treatment. Information about all SAEs is collected and recorded on the **Serious Adverse Event Report Form**; all applicable sections of the form must be completed in order to provide a clinically thorough report. The investigator must assess and record the relationship of each SAE to each specific study treatment (if there is more than one study treatment), complete the SAE Report Form, and submit the completed form.
Each reoccurrence, complication, or progression of the original event should be reported as a follow-up to that event regardless of when it occurs. The follow-up information should describe whether the event has resolved or continues, if and how it was treated, whether the blind was broken or not, and whether the patient continued or withdrew from study participation.
An investigator who is a qualified physician will evaluate all adverse events according to the NCI Common Terminology for Adverse Events (CTCAE), version 5.0. Any adverse event which changes CTCAE grade over the course of a given episode will have each change of grade recorded on the adverse event case report forms/worksheets. All adverse events regardless of CTCAE grade must also be evaluated for seriousness.

Second and secondary malignancy

A *secondary malignancy* is a cancer caused by treatment for a previous malignancy (*e.g.*, treatment with investigational agent/intervention, radiation or chemotherapy). A secondary malignancy is not considered a metastasis of the initial neoplasm.
All secondary malignancies that occur following treatment with an agent under an IND/IDE must be reported through **ONCORE.**
Three options are available to describe the event:

- Leukemia secondary to oncology chemotherapy (*e.g.*, acute myelocytic leukemia [AML])
- Myelodysplastic syndrome (MDS)
- Treatment-related secondary malignancy

Any malignancy possibly related to cancer treatment (including AML/MDS) should also be reported via the routine reporting mechanisms outlined in each protocol.
A second malignancy is one unrelated to the treatment of a prior malignancy (and is **NOT** a metastasis from the initial malignancy).

Definition of unanticipated problems (UP) and reporting requirements

The Office for Human Research Protections (OHRP) considers unanticipated problems involving risks to participants or others to include, in general, any incident, experience, or an outcome that meets **all** the following criteria: Unexpected in terms of nature, severity, or frequency given (a) the research procedures that are described in the protocol-related documents, such as the IRB-approved research protocol and informed consent document; and (b) the characteristics of the participant population being studied; Related or possibly related to participation in the research ("possibly related" means there is a reasonable possibility that the incident, experience, or outcome may have been caused by the procedures involved in the research); and Suggests that the research places participants or others at a greater risk of harm (including physical, psychological, economic, or social harm) than was previously known or recognized. This study will use the OHRP definition of unanticipated problems. Incidents or events that meet the OHRP criteria for UPs require the creation and completion of a UP report form. It is the site investigator's responsibility to report UPs to their IRB and to the DCC/study sponsor. The UP report will include the following information: Protocol identifying information: protocol title and number, PI's name, and the IRB project number; A detailed description of the event, incident, experience, or

outcome; An explanation of the basis for determining that the event, incident, experience, or outcome represents an UP; A description of any changes to the protocol or other corrective actions that have been taken or are proposed in response to the UP. We will make an assessment of whether the event constitutes an unanticipated problem posing risks to subjects or others (UP). This assessment will be provided to the Emory University IRB. If the Emory IRB determines an event is a UP it will notify the appropriate regulatory agencies and institutional officials.

The Data and Safety Monitoring Committee (DSMC)

The Data and Safety Monitoring Committee (DSMC) of the Winship Cancer Institute will provide oversight for the conduct of this study. The DSMC functions independently within Winship Cancer Institute to conduct internal monitoring functions to ensure that research being conducted by Winship Cancer Institute Investigators produces high-quality scientific data in a manner consistent with good clinical practice (GCP) and appropriate regulations that govern clinical research. Depending on the risk level of the protocol, the DSMC review may occur every 6 months or annually. For studies deemed High Risk, initial study monitoring will occur within 6 months from the date of the first subject accrued, with 2 of the first 5 subjects being reviewed. For studies deemed Moderate Risk, initial study monitoring will occur within 1 year from the date of the first subject accrued, with 2 of the first 5 subjects being reviewed. Subsequent monitoring and data analysis will occur in routine intervals per the Winship Data and Safety Monitoring Plan (DSMP): 6 months for a high risk study and 1 year for a moderate risk study. The DSMC will review pertinent aspects of the study to assess subject safety, compliance with the protocol, data collection, and risk-benefit ratio. Specifically, the Winship Cancer Institute Internal Monitors assigned to the DSMC may verify informed consent, eligibility, data entry, accuracy and availability of source documents, AEs/SAEs, and essential regulatory documents. Following the monitoring review, monitors will provide a preliminary report of monitoring findings to the PI and other pertinent individuals involved in the conduct of the study. The PI is required to address and respond to all the deficiencies noted in the preliminary report. Prior to the completion of the final summary report, monitors will discuss the preliminary report responses with the PI and other team members (when appropriate). A final monitoring summary report will then be prepared by the monitor. Final DSMC review will include the final monitoring summary report with corresponding PI response, submitted CAPA (when applicable), PI Summary statement, and available aggregate toxicity and safety data. The DSMC will render a recommendation and rating based on the overall trial conduct. The PI is responsible for ensuring that instances of egregious data insufficiencies are reported to the IRB. Continuing Review submissions will include the DSMC recommendation letter. Should any revisions be made to the protocol-specific monitoring plan after initial DSMC approval, the PI will be responsible for notifying the DSMC of such changes. The Committee reserves the right to conduct additional audits if necessary. The PI will carryout study oversight by monthly working group meetings following the scheduled data analysis, random chart reviews and meeting with study team members as appropriate. The study team will be trained on all relevant study procedures with an initial meeting at the time of study approval with the PI and all co-investigators. Additionally, as new team members will be trained on the protocol before being added to the study team.

17. Provisions to Protect the Privacy Interests of Participants

Participants will be assured of their voluntary participation in the study, their choice to answer or not answer any question, and the protocol for maintaining confidentiality.

Participant confidentiality is strictly held in trust by the participating investigators, their staff, and the sponsor(s) and their agents. This confidentiality is extended to cover testing of biological samples and genetic tests in addition to the clinical information relating to participants. Therefore, the study protocol, documentation, data, and all other information generated will be held in strict confidence. No information

concerning the study or the data will be released to any unauthorized third party without prior written approval of the sponsor.

The study monitor, other authorized representatives of the sponsor, representatives of the IRB or pharmaceutical company supplying study product may inspect all documents and records required to be maintained by the investigator, including but not limited to, medical records (office, clinic, or hospital) and pharmacy records for the participants in this study. The clinical study site will permit access to such records.

The study participant's contact information will be securely stored at each clinical site for internal use during the study. At the end of the study, all records will continue to be kept in a secure location for as long a period as dictated by local IRB and Institutional regulations.

Study participant research data, which is for purposes of statistical analysis and scientific reporting, will be transmitted to and stored. This will not include the participant's contact or identifying information. Rather, individual participants and their research data will be identified by a unique study identification number. The study data entry and study management systems used by clinical sites and research staff will be secured and password protected. At the end of the study, all study databases will be de-identified and archived

18. Economic Burden to Participants

If subjects have insurance, Emory will submit claims to the insurance for items and services that the sponsor does not cover. Emory will send in only those claims for items and services that it reasonably believes the insurance will pay and that the sponsor has not paid. The actual amount that participants have to pay depends on whether or not they have health insurance and whether or not that insurance will pay for any research study costs. Generally, insurance companies will not pay for items and services that are required just for a research study. Some insurance companies will not pay for regular medical treatment or treatment for complications if in a study. If subject do not have insurance, Emory will review that particular case as part of its program for low-income patient care. The standard policies of that program will apply. The program will figure out if subjects have to pay any costs for taking part in the study and what those costs will be

19. Consent Process

The initial informed consent discussion will occur in Winship Cancer Institute or the Emory Clinic.
At Winship Cancer Institute, the informed consent is an ongoing, interactive process rather than a one-time information session. The consent form document is designed to begin the informed consent process, which provides the patient with ongoing explanations that will help them make educational decisions about whether to begin or continue participating in the trial. The research team knows that a written document alone may not ensure that the patient fully understands what participation means. Therefore, the research team will discuss with the patient the trial's purpose, procedures, risks and potential benefits, and their rights as a participant. The team will continue to update the patient on any new information that may affect their situation.
Consent will be obtained prior to any research-driven procedures. The investigator will assess the patient's capacity during his/her encounters with him or her. The investigator will give the person providing consent adequate opportunity to read the consent document before it is signed and dated.
It will be explained to prospective participants that the study involves research, the purpose of the research, the expected duration of participation, as well as the approximate number of participants to be enrolled. The study procedures, and identification of research procedures v. non-research will also be thoroughly discussed. It will be explained to participants that participation is voluntary and that the subject may discontinue at any time.

Refusal to participate or withdraw will not involve a penalty or loss of benefits to which the participant is otherwise entitled. Refusal will in no way affect the participant's future are. The participant will also be told of the possible consequences of the decision to withdraw from the research, and procedures for orderly termination of participation.

Any significant new findings developed during the course of the research that may affect the participant's willingness to continue to participate will be provided. Also explained will be anticipated circumstances under which the subject's participation may be terminated by the investigator without the participant's consent.

Prospective participants will be provided with a description of any reasonably forseeable risks or discomforts as well as a description of any benefits to the participant or to others that might be reasons expected from the research. Alternative procedures or courses of treatment will also be thoroughly discussed.

Prospective participants will also be given detailed information describing the extent to which confidentiality of records identifying the participant will be maintained and what records may be examined by the research staff, IRBs, sponsor, their representatives, and possibly the FDA or OHRP.

Also communicated to the participant will be an explanation that emergency medical care will be arranged for a study-related illness or injury, and an explanation of whether funds are set aside to pay for this care and/or compensation, and if so by whom (e.g., sponsor, subject, insurer). The participant is told the source of the study's funding.

All participants will be told of any additional costs that may result from participation in the research.

Non-English-Speaking Participants

A certified translator/interpreter will be present during the consenting process and all questions and concerns will be answered by the treating physician.

A Short Form in that specific language will be used. A certified translator/interpreter will be present during the consenting process and this will be documented. We will use what's available on Emory IRB website. For the languages that are not available, we will have the short form translated to that language and submit the IRB for review and approval prior to use. Process to Document Consent in Writing: Winship SOP 2.1:"Obtaining Informed consent for Interventional clinical trial" will be followed.

Participants who are not yet adults (infants, children, teenagers)

n/a

Cognitively Impaired Adults

n/a

Adults Unable to Consent

n/a

20. Setting

The research will be conducted at Emory University:

Potential participants will be identified in neurosurgery clinics, multidisciplinary cancer clinic, surgical oncology clinics, in-patient wards, multidisciplinary tumor board at Emory University.

21. Resources Available

Emory University was founded in 1836 and is a national center for teaching, research, and service. Emory University has been named as one of the nation's top 25 universities for more than a decade by the U.S. News and World Report. Emory University research partners include the Georgia Institute of Technology, the University of Georgia, Morehouse School of Medicine, the US Centers for Disease Control and Prevention, Children's Healthcare of Atlanta, and the Georgia Clinical and Translational Science Alliance (GACTSA). Emory University researchers received $734 million from external funding agencies in fiscal year 2018, including approximately $441 million in funding from federal agencies, $359 million of this from the National Institutes of Health (NIH).

Winship Cancer Institute (Winship) is Georgia's first and only National Cancer Institute (NCI)-designated Comprehensive Cancer Center (P30CA138292) and is dedicated to the integration of innovative clinical and basic science research with outstanding patient care for the prevention, treatment and control of cancer. First designated in 2009, Winship's NCI designation was renewed in 2012 and 2016, achieving an "outstanding" rating. Winship earned the prestigious Comprehensive Cancer Center designation from the NCI in 2016, after demonstrating that its outstanding programs are reducing the cancer burden on the state of Georgia through research conducted in its laboratories, its clinical trial program, and its population-based science. The institutional support for Winship was rated as 'exceptional' by the review panel.

The **Winship Clinic Building C** houses the primary offices and clinical space for cancer services including the medical oncology, hematology, and surgical oncology clinics, the radiation oncology program, and the Winship Ambulatory Infusion Center. In summer 2017, Emory Healthcare completed the expansion of **Emory University Hospital Tower** on Clifton Road. This nine-floor facility adds 144 inpatient beds to the hospital, of which more than 80% are dedicated to cancer care. The hospital expansion also accommodates cancer patient-specific intensive care units, an expanded BMT Unit with peri-transplant clinics to facilitate continuity of care, and a 24-hour cancer urgent care center, which serves as both a triage facility and short stay treatment center for patients with cancer-related medical concerns.

The **Winship Phase I Unit**, on the fourth floor of the Emory University Hospital Tower, is the largest unit in Georgia dedicated to the earliest and most critical phase of new cancer therapy evaluation. There is space for 15 private treatment bays, four clinic rooms, its own lab for doing patient blood work, a dedicated secure medication room, computer workspace for research and other support staff, and a "fast track" bay with three chairs for rapid use in patients who, for example, might need only a research lab test done.

16. References

1. Jansen CS, Prokhnevska N, Master VA, et al. An intra-tumoral niche maintains and differentiates stem-like CD8 T cells. Nature 2019;576:465-70.
2. Aoyama H, Shirato H, Tago M, et al. Stereotactic radiosurgery plus whole-brain radiation therapy vs stereotactic radiosurgery alone for treatment of brain metastases: a randomized controlled trial. Jama 2006;295:2483-91.
3. Brown PD, Jaeckle K, Ballman KV, et al. Effect of Radiosurgery Alone vs Radiosurgery With Whole Brain Radiation Therapy on Cognitive Function in Patients With 1 to 3 Brain Metastases: A Randomized Clinical Trial. JAMA 2016;316:401-9.
4. Chang EL, Wefel JS, Hess KR, et al. Neurocognition in patients with brain metastases treated with radiosurgery or radiosurgery plus whole-brain irradiation: a randomised controlled trial. Lancet Oncol 2009;10:1037-44.

5. Kocher M, Soffietti R, Abacioglu U, et al. Adjuvant whole-brain radiotherapy versus observation after radiosurgery or surgical resection of one to three cerebral metastases: results of the EORTC 22952-26001 study. J Clin Oncol 2011;29:134-41.
6. Mahajan A, Ahmed S, McAleer MF, et al. Post-operative stereotactic radiosurgery versus observation for completely resected brain metastases: a single-centre, randomised, controlled, phase 3 trial. Lancet Oncol 2017;18:1040-8.
7. Patchell RA, Tibbs PA, Regine WF, et al. Postoperative radiotherapy in the treatment of single metastases to the brain: a randomized trial. JAMA 1998;280:1485-9.
8. Asher AL, Burri SH, Wiggins WF, et al. A new treatment paradigm: neoadjuvant radiosurgery before surgical resection of brain metastases with analysis of local tumor recurrence. International journal of radiation oncology, biology, physics 2014;88:899-906.
9. Patel KR, Burri SH, Asher AL, et al. Comparing Preoperative With Postoperative Stereotactic Radiosurgery for Resectable Brain Metastases: A Multi-institutional Analysis. Neurosurgery 2016;79:279-85.
10. Patel KR, Burri SH, Boselli D, et al. Comparing pre-operative stereotactic radiosurgery (SRS) to post-operative whole brain radiation therapy (WBRT) for resectable brain metastases: a multi-institutional analysis. Journal of neuro-oncology 2017;131:611-8.
11. Prabhu RS, Miller KR, Asher AL, et al. Preoperative stereotactic radiosurgery before planned resection of brain metastases: updated analysis of efficacy and toxicity of a novel treatment paradigm. J Neurosurg 2018:1-8.
12. Brown PD, Ballman KV, Cerhan JH, et al. Postoperative stereotactic radiosurgery compared with whole brain radiotherapy for resected metastatic brain disease (NCCTG N107C/CEC.3): a multicentre, randomised, controlled, phase 3 trial. Lancet Oncol 2017;18:1049-60.
13. Brown PD, Jaeckle K, Ballman KV, et al. Effect of Radiosurgery Alone vs Radiosurgery With Whole Brain Radiation Therapy on Cognitive Function in Patients With 1 to 3 Brain Metastases: A Randomized Clinical Trial. JAMA 2016;316:401-9.
14. Press RH, Zhang C, Cassidy RJ, et al. Targeted sequencing and intracranial outcomes of patients with lung adenocarcinoma brain metastases treated with radiotherapy. Cancer 2018;124:3586-95.
15. Ayala-Peacock DN, Peiffer AM, Lucas JT, et al. A nomogram for predicting distant brain failure in patients treated with gamma knife stereotactic radiosurgery without whole brain radiotherapy. Neuro Oncol 2014;16:1283-8.
16. Press RH, Boselli DM, Symanowski JT, et al. External Validity of a Risk Stratification Score Predicting Early Distant Brain Failure and Salvage Whole Brain Radiation Therapy After Stereotactic Radiosurgery for Brain Metastases. Int J Radiat Oncol Biol Phys 2017;98:632-8.
17. Dudnik E, Yust-Katz S, Nechushtan H, et al. Intracranial response to nivolumab in NSCLC patients with untreated or progressing CNS metastases. Lung Cancer 2016;98:114-7.
18. Goldberg SB, Gettinger SN, Mahajan A, et al. Pembrolizumab for patients with melanoma or non-small-cell lung cancer and untreated brain metastases: early analysis of a non-randomised, open-label, phase 2 trial. Lancet Oncol 2016;17:976-83.
19. Hendriks LEL, Henon C, Auclin E, et al. Outcome of Patients with Non-Small Cell Lung Cancer and Brain Metastases Treated with Checkpoint Inhibitors. J Thorac Oncol 2019;14:1244-54.
20. Naito Y, Saito K, Shiiba K, et al. CD8+ T cells infiltrated within cancer cell nests as a prognostic factor in human colorectal cancer. Cancer Res 1998;58:3491-4.
21. Sharma P, Shen Y, Wen S, et al. CD8 tumor-infiltrating lymphocytes are predictive of survival in muscle-invasive urothelial carcinoma. Proc Natl Acad Sci U S A 2007;104:3967-72.

22. Hiraoka K, Miyamoto M, Cho Y, et al. Concurrent infiltration by CD8+ T cells and CD4+ T cells is a favourable prognostic factor in non-small-cell lung carcinoma. Br J Cancer 2006;94:275-80.
23. Dieci MV, Tsvetkova V, Orvieto E, et al. Immune characterization of breast cancer metastases: prognostic implications. Breast Cancer Res 2018;20:62.
24. Harter PN, Bernatz S, Scholz A, et al. Distribution and prognostic relevance of tumor-infiltrating lymphocytes (TILs) and PD-1/PD-L1 immune checkpoints in human brain metastases. Oncotarget 2015;6:40836-49.
25. Berghoff AS, Ricken G, Wilhelm D, et al. Tumor infiltrating lymphocytes and PD-L1 expression in brain metastases of small cell lung cancer (SCLC). J Neurooncol 2016;130:19-29.
26. Binnewies M, Roberts EW, Kersten K, et al. Understanding the tumor immune microenvironment (TIME) for effective therapy. Nat Med 2018;24:541-50.
27. Sautes-Fridman C, Petitprez F, Calderaro J, Fridman WH. Tertiary lymphoid structures in the era of cancer immunotherapy. Nat Rev Cancer 2019;19:307-25.
28. Im SJ, Hashimoto M, Gerner MY, et al. Defining CD8+ T cells that provide the proliferative burst after PD-1 therapy. Nature 2016;537:417-21.
29. Scott SC, Pennell NA. Early Use of Systemic Corticosteroids in Patients with Advanced NSCLC Treated with Nivolumab. J Thorac Oncol 2018;13:1771-5.
30. Arbour KC, Mezquita L, Long N, et al. Impact of Baseline Steroids on Efficacy of Programmed Cell Death-1 and Programmed Death-Ligand 1 Blockade in Patients With Non-Small-Cell Lung Cancer. J Clin Oncol 2018;36:2872-8.
31. Silina K, Soltermann A, Attar FM, et al. Germinal Centers Determine the Prognostic Relevance of Tertiary Lymphoid Structures and Are Impaired by Corticosteroids in Lung Squamous Cell Carcinoma. Cancer Res 2018;78:1308-20.
32. Buchwald ZNTL, J; Eberhardt, CS; Wieland, A; Im SJ; Lawson, D; Curran, W; Ahmed, R; Khan, MK. The tumor-draining lymph node is important for a robust abscopal effect stimulated by radiotherapy. Journal for ImmunoTherapy of Cancer; Accepted 2020.
33. Twyman-Saint Victor C, Rech AJ, Maity A, et al. Radiation and dual checkpoint blockade activate non-redundant immune mechanisms in cancer. Nature 2015;520:373-7.
34. Abuodeh Y, Venkat P, Kim S. Systematic review of case reports on the abscopal effect. Curr Probl Cancer 2016;40:25-37.
35. Wersall PJ, Blomgren H, Pisa P, Lax I, Kalkner KM, Svedman C. Regression of non-irradiated metastases after extracranial stereotactic radiotherapy in metastatic renal cell carcinoma. Acta Oncol 2006;45:493-7.
36. Kingsley DP. An interesting case of possible abscopal effect in malignant melanoma. Br J Radiol 1975;48:863-6.
37. Vatner RE, Cooper BT, Vanpouille-Box C, Demaria S, Formenti SC. Combinations of immunotherapy and radiation in cancer therapy. Front Oncol 2014;4:325.
38. Buchwald ZS, Wynne J, Nasti TH, et al. Radiation, Immune Checkpoint Blockade and the Abscopal Effect: A Critical Review on Timing, Dose and Fractionation. Front Oncol 2018;8:612.
39. Theelen W, Peulen HMU, Lalezari F, et al. Effect of Pembrolizumab After Stereotactic Body Radiotherapy vs Pembrolizumab Alone on Tumor Response in Patients With Advanced Non-Small Cell Lung Cancer: Results of the PEMBRO-RT Phase 2 Randomized Clinical Trial. JAMA Oncol 2019.
40. McBride S, Sherman E, Tsai CJ, et al. Randomized Phase II Trial of Nivolumab With Stereotactic Body Radiotherapy Versus Nivolumab Alone in Metastatic Head and Neck Squamous Cell Carcinoma. J Clin Oncol 2020:JCO2000290.

41. Luke JJ, Lemons JM, Karrison TG, et al. Safety and Clinical Activity of Pembrolizumab and Multisite Stereotactic Body Radiotherapy in Patients With Advanced Solid Tumors. J Clin Oncol 2018;36:1611-8.

42. Prabhu RS, Press RH, Patel KR, et al. Single-Fraction Stereotactic Radiosurgery (SRS) Alone Versus Surgical Resection and SRS for Large Brain Metastases: A Multi-institutional Analysis. Int J Radiat Oncol Biol Phys 2017;99:459-67.

43. Shaw E, Scott C, Souhami L, et al. Single dose radiosurgical treatment of recurrent previously irradiated primary brain tumors and brain metastases: final report of RTOG protocol 90-05. Int J Radiat Oncol Biol Phys 2000;47:291-8.

APPENDIX A PERFORMANCE STATUS CRITERIA

**ECOG Performance Status Scale**		**Karnofsky Performance Scale**	
Grade	Descriptions	Percent	Description
0	Normal activity. Fully active, able to carry on all pre-disease performance without restriction.	100	Normal, no complaints, no evidence of disease.
		90	Able to carry on normal activity; minor signs or symptoms of disease.
1	Symptoms, but ambulatory. Restricted in physically strenuous activity, but ambulatory and able to carry out work of a light or sedentary nature (*e.g.*, light housework, office work).	80	Normal activity with effort; some signs or symptoms of disease.
		70	Cares for self, unable to carry on normal activity or to do active work.
2	In bed <50% of the time. Ambulatory and capable of all self-care, but unable to carry out any work activities. Up and about more than 50% of waking hours.	60	Requires occasional assistance, but is able to care for most of his/her needs.
		50	Requires considerable assistance and frequent medical care.
3	In bed >50% of the time. Capable of only limited self-care, confined to bed or chair more than 50% of waking hours.	40	Disabled, requires special care and assistance.
		30	Severely disabled, hospitalization indicated. Death not imminent.
4	100% bedridden. Completely disabled. Cannot carry on any self-care. Totally confined to bed or chair.	20	Very sick, hospitalization indicated. Death not imminent.
		10	Moribund, fatal processes progressing rapidly.
5	Dead.	0	Dead.

As published in Am. J. Clin. Oncol.: *Oken, M.M., Creech, R.H., Tormey, D.C., Horton, J., Davis, T.E., McFadden, E.T., Carbone, P.P.: Toxicity And Response Criteria Of The Eastern Cooperative Oncology Group. Am J Clin Oncol 5:649-655, 1982.* The Eastern Cooperative Oncology Group, Robert Comis M.D., Group Chair

APPENDIX B Abbreviations and definition of terms

The following abbreviations and special terms are used in this study Clinical Study Protocol.

Abbreviation or special term	Explanation
ADA	Anti-drug antibody
AE	Adverse event
AESI	Adverse event of special interest
ALK	Anaplastic lymphoma kinase
ALT	Alanine aminotransferase
APF12	Proportion of patients alive and progression free at 12 months from randomization
AST	Aspartate aminotransferase
BoR	Best objective response
BP	Blood pressure
C	Cycle
CD	Cluster of differentiation
CI	Confidence interval
CL	Clearance
C_{max}	Maximum plasma concentration
$C_{max,ss}$	Maximum plasma concentration at steady state
CR	Complete response
CSA	Clinical study agreement
CSR	Clinical study report
CT	Computed tomography
CTCAE	Common Terminology Criteria for Adverse Event
CTLA-4	Cytotoxic T–lymphocyte-associated antigen 4
$C_{trough,ss}$	Trough concentration at steady state
CXCL	Chemokine (C-X-C motif) ligand
DoR	Duration of response
EC	Ethics Committee, synonymous to Institutional Review Board and Independent Ethics Committee
ECG	Electrocardiogram
ECOG	Eastern Cooperative Oncology Group
eCRF	Electronic case report form

Abbreviation or special term	Explanation
EDoR	Expected duration of response
EGFR	Epidermal growth factor receptor
EU	European Union
FAS	Full analysis set
FDA	Food and Drug Administration
GCP	Good Clinical Practice
GI	Gastrointestinal
GMP	Good Manufacturing Practice
hCG	Human chorionic gonadotropin
HIV	Human immunodeficiency virus
HR	Hazard ratio
IB	Investigator’s Brochure
ICF	Informed consent form
ICH	International Conference on Harmonisation
IDMC	Independent Data Monitoring Committee
IFN	Interferon
IgE	Immunoglobulin E
IgG	Immunoglobulin G
IHC	Immunohistochemistry
IL	Interleukin
ILS	Interstitial lung disease
IM	Intramuscular
IMT	Immunomodulatory therapy
IP	Investigational product
irAE	Immune-related adverse event
IRB	Institutional Review Board
irRECIST	Immune-related Response Evaluation Criteria in Solid Tumors
ITT	Intent-to-Treat
IV	Intravenous
IVRS	Interactive Voice Response System
IWRS	Interactive Web Response System
mAb	Monoclonal antibody
MDSC	Myeloid-derived suppressor cell

Abbreviation or special term	Explanation
MedDRA	Medical Dictionary for Regulatory Activities
MHLW	Minister of Health, Labor, and Welfare
miRNA	Micro-ribonucleic acid
MRI	Magnetic resonance imaging
NCI	National Cancer Institute
NE	Not evaluable
NSCLC	Non–small-cell lung cancer
OAE	Other significant adverse event
ORR	Objective response rate
OS	Overall survival
PBMC	Peripheral blood mononuclear cell
PD	Progressive disease
PDx	Pharmacodynamic(s)
PFS	Progression-free survival
PFS2	Time to second progression
PGx	Pharmacogenetic research
PK	Pharmacokinetic(s)
PR	Partial response
q2w	Every 2 weeks
q3w	Every 3 weeks
q4w	Every 4 weeks
q6w	Every 6 weeks
q8w	Every 8 weeks
QTcF	QT interval corrected for heart rate using Fridericia's formula
RECIST 1.1	Response Evaluation Criteria in Solid Tumors, version 1.1
RNA	Ribonucleic acid
RR	Response rate
RT-QPCR	Reverse transcription quantitative polymerase chain reaction
SAE	Serious adverse event
SAP	Statistical analysis plan
SAS	Safety analysis set
SCLC	Small cell lung cancer
SD	Stable disease

Abbreviation or special term	Explanation
SNP	Single nucleotide polymorphism
SoC	Standard of Care
T_3	Triiodothyronine
T_4	Thyroxine
TSH	Thyroid-stimulating hormone
ULN	Upper limit of normal
US	United States
WBDC	Web-Based Data Capture
WHO	World Health Organization
