# Supplementary material for: Pre-operative stereotactic radiosurgery and peri-operative dexamethasone for resectable brain metastases: a two-arm pilot study evaluating clinical outcomes and immunological correlates
Source: Nat Commun. 2024 Oct 14;15:8854. doi: 10.1038/s41467-024-53034-6 (PMC11473782; doi:10.1038/s41467-024-53034-6)
Supplement: Supplementary file 3 — Reporting Summary [file 41467_2024_53034_MOESM3_ESM.pdf]

nature portfolio

Corresponding author(s): Zachary Buchwald; Haydn Kissick

Last updated by author(s): Jul 19, 2024

Reporting Summary

Nature Portfolio wishes to improve the reproducibility of the work that we publish. This form provides structure for consistency and transparency in reporting. For further information on Nature Portfolio policies, see our Editorial Policies and the Editorial Policy Checklist.

Statistics

For all statistical analyses, confirm that the following items are present in the figure legend, table legend, main text, or Methods section.

n/a	Confirmed	
[ ]	[x]	The exact sample size (*n*) for each experimental group/condition, given as a discrete number and unit of measurement
[ ]	[x]	A statement on whether measurements were taken from distinct samples or whether the same sample was measured repeatedly
[ ]	[x]	The statistical test(s) used AND whether they are one- or two-sided<br>*Only common tests should be described solely by name; describe more complex techniques in the Methods section.*
[ ]	[x]	A description of all covariates tested
[x]	[ ]	A description of any assumptions or corrections, such as tests of normality and adjustment for multiple comparisons
[ ]	[x]	A full description of the statistical parameters including central tendency (e.g. means) or other basic estimates (e.g. regression coefficient) AND variation (e.g. standard deviation) or associated estimates of uncertainty (e.g. confidence intervals)
[ ]	[x]	For null hypothesis testing, the test statistic (e.g. *F*, *t*, *r*) with confidence intervals, effect sizes, degrees of freedom and *P* value noted<br>*Give P values as exact values whenever suitable.*
[x]	[ ]	For Bayesian analysis, information on the choice of priors and Markov chain Monte Carlo settings
[x]	[ ]	For hierarchical and complex designs, identification of the appropriate level for tests and full reporting of outcomes
[x]	[ ]	Estimates of effect sizes (e.g. Cohen's *d*, Pearson's *r*), indicating how they were calculated

Our web collection on statistics for biologists contains articles on many of the points above.

Software and code

Policy information about availability of computer code

Data collection	*Provide a description of all commercial, open source and custom code used to collect the data in this study, specifying the version used OR state that no software was used.*
Data analysis	Cellranger v3.1, VISION R package V2.1

For manuscripts utilizing custom algorithms or software that are central to the research but not yet described in published literature, software must be made available to editors and reviewers. We strongly encourage code deposition in a community repository (e.g. GitHub). See the Nature Portfolio guidelines for submitting code & software for further information.

Data

Policy information about availability of data

All manuscripts must include a data availability statement. This statement should provide the following information, where applicable:

- Accession codes, unique identifiers, or web links for publicly available datasets
- A description of any restrictions on data availability
- For clinical datasets or third party data, please ensure that the statement adheres to our policy

Source data and study protocol provided with the paper. scRNA-seq data are available in the NCBI Gene Expression Omnibus (GEO) database under the accession number GSE275929.

Research involving human participants, their data, or biological material

Policy information about studies with human participants or human data. See also policy information about sex, gender (identity/presentation), and sexual orientation and race, ethnicity and racism.

Reporting on sex and gender	Sex is reported in the manuscript however with numbers of males and females on both arms of the propsective clinical trial. Subgroup analysis was not performed due to low N's.
Reporting on race, ethnicity, or other socially relevant groupings	*Please specify the socially constructed or socially relevant categorization variable(s) used in your manuscript and explain why they were used. Please note that such variables should not be used as proxies for other socially constructed/relevant variables (for example, race or ethnicity should not be used as a proxy for socioeconomic status).* *Provide clear definitions of the relevant terms used, how they were provided (by the participants/respondents, the researchers, or third parties), and the method(s) used to classify people into the different categories (e.g. self-report, census or administrative data, social media data, etc.)* *Please provide details about how you controlled for confounding variables in your analyses.*
Population characteristics	Relevant co-variates including age, performance status, pre-op SRS volume and pre-op SRS dose were all recorded in Table 1 and were not significantly different between arms.
Recruitment	Patient's presenting or admitted to Emory University Hospital with newly diagnosed brain metastases were referred to radiation oncology and the trial was discussed with them in detail.
Ethics oversight	Emory University Institutional Review Board

Note that full information on the approval of the study protocol must also be provided in the manuscript.

Field-specific reporting

Please select the one below that is the best fit for your research. If you are not sure, read the appropriate sections before making your selection.

[x] Life sciences   [ ] Behavioural & social sciences   [ ] Ecological, evolutionary & environmental sciences

For a reference copy of the document with all sections, see nature.com/documents/nr-reporting-summary-flat.pdf

Life sciences study design

All studies must disclose on these points even when the disclosure is negative.

Sample size	Samples size was determined based on pre-specified power calculation to detect a toxicity rate at 4-months post-SRS.
Data exclusions	No data that was acquired was excluded.
Replication	Patients were all included. Two datasets in this study were used a prospectively collected and retrospectively analyzed which showed similar results. Relevant co-variates were included in all performed multivariate analyses.
Randomization	No randomization was used
Blinding	No blinding was used in this study as it was impractical given the main investigator was managing all the medication and SRS treatment.

Reporting for specific materials, systems and methods

We require information from authors about some types of materials, experimental systems and methods used in many studies. Here, indicate whether each material, system or method listed is relevant to your study. If you are not sure if a list item applies to your research, read the appropriate section before selecting a response.

Materials & experimental systems

n/a	Involved in the study	
[ ]	[x]	Antibodies
[x]	[ ]	Eukaryotic cell lines
[x]	[ ]	Palaeontology and archaeology
[x]	[ ]	Animals and other organisms
[ ]	[x]	Clinical data
[x]	[ ]	Dual use research of concern
[x]	[ ]	Plants

Methods

n/a	Involved in the study	
[x]	[ ]	ChIP-seq
[ ]	[x]	Flow cytometry
[x]	[ ]	MRI-based neuroimaging

Antibodies

Antibodies used	Listed in Table 7 of the manuscript.
Validation	All antibodies were validated in previous publications. See Jansen et al. Nature. 2019; Prokhnevska et al Immunity 2023

Clinical data

Policy information about clinical studies

All manuscripts should comply with the ICMJEguidelines for publication of clinical research and a completedCONSORT checklist must be included with all submissions.

Clinical trial registration	NCT04895592
Study protocol	Included with paper
Data collection	All prospective data was all collected at Emory University; retrospective data was collected at Emory University Winship Cancer Institute and the Levine Cancer Institute in Charlotte, NC.

Outcomes

Primary outcomes was a prespecified safety endpoint: The primary objective of this study is to describe the safety in terms of grade > 3 CNS toxicity at 4 months. Tolerability of this regimen will be defined as <33% of patients develop grade > 3 at 4 months. This interval is based on (1) unusual radiation related adverse effects beginning as early as 4 months post SRS, and (2) typical timeline used in other trials.

Niche Density: Niche density will be assessed by immunofluorescence as described above. It will be summarized descriptively as the percent of tumor occupied by immune niche for each treatment arm, and will be compared between Arms A and B using a two-sample t-test or non-parametric equivalent, such as Mann-Whitney U test.

Time to Local Recurrence (LR): Time to LR is defined as time from pre-operative SRS to intracranial progression at the treated site. Those without intracranial progression at the treated site will be censored at last follow-up date or death. LR will be estimated using the Kaplan-Meier method, and a 95% confidence interval for median LR will be estimated using the Brookmeyer-Crowley approach.

Time to Distant Brain Failure is defined as time from pre-operative SRS to intracranial progression at any site within the brain.

Overall survival (OS): Overall survival is defined as time from pre-operative SRS initiation to death. Those alive will be censored at date of last follow-up. OS will be estimated using the Kaplan-Meier method, and a 95% confidence interval for median OS will be estimated using the Brookmeyer-Crowley approach.

Plants

Seed stocks	*Report on the source of all seed stocks or other plant material used. If applicable, state the seed stock centre and catalogue number. If plant specimens were collected from the field, describe the collection location, date and sampling procedures.*
Novel plant genotypes	*Describe the methods by which all novel plant genotypes were produced. This includes those generated by transgenic approaches, gene editing, chemical/radiation-based mutagenesis and hybridization. For transgenic lines, describe the transformation method, the number of independent lines analyzed and the generation upon which experiments were performed. For gene-edited lines, describe the editor used, the endogenous sequence targeted for editing, the targeting guide RNA sequence (if applicable) and how the editor was applied.*
Authentication	*Describe any authentication procedures for each seed stock used or novel genotype generated. Describe any experiments used to assess the effect of a mutation and, where applicable, how potential secondary effects (e.g. second site T-DNA insertions, mosiacism, off-target gene editing) were examined.*

Flow Cytometry

Plots

Confirm that:

- [x] The axis labels state the marker and fluorochrome used (e.g. CD4-FITC).
- [x] The axis scales are clearly visible. Include numbers along axes only for bottom left plot of group (a 'group' is an analysis of identical markers).
- [x] All plots are contour plots with outliers or pseudocolor plots.
- [x] A numerical value for number of cells or percentage (with statistics) is provided.

Methodology

Sample preparation	Standard processing with tumor digestion and then density gradient as described in the manuscript.
Instrument	BD FACSymphony
Software	Flowjo v10
Cell population abundance	*Describe the abundance of the relevant cell populations within post-sort fractions, providing details on the purity of the samples and how it was determined.*
Gating strategy	Gating strategy is shown in Supplemental figures

☒ Tick this box to confirm that a figure exemplifying the gating strategy is provided in the Supplementary Information.
